# Global metagenomic survey reveals a new bacterial candidate phylum in geothermal springs

**DOI:** 10.1038/ncomms10476

**Published:** 2016-01-27

**Authors:** Emiley A. Eloe-Fadrosh, David Paez-Espino, Jessica Jarett, Peter F. Dunfield, Brian P. Hedlund, Anne E. Dekas, Stephen E. Grasby, Allyson L. Brady, Hailiang Dong, Brandon R. Briggs, Wen-Jun Li, Danielle Goudeau, Rex Malmstrom, Amrita Pati, Jennifer Pett-Ridge, Edward M. Rubin, Tanja Woyke, Nikos C. Kyrpides, Natalia N. Ivanova

**Affiliations:** 1 Department of Energy Joint Genome Institute, Walnut Creek, California 94598, USA; 2 Department of Biological Sciences, University of Calgary, Calgary, Alberta T2N 1N4, Canada; 3 School of Life Sciences, University of Nevada, Las Vegas, Las Vegas, Nevada 89154, USA; 4 Lawrence Livermore National Laboratory, Livermore, California 94550, USA; 5 Geological Survey of Canada, Calgary, Alberta T2L 2A7, Canada; 6 School of Geography & Earth Sciences, McMaster University, Hamilton, Ontario L8S 4L8, Canada; 7 Department of Geology and Environmental Earth Sciences, Miami University, Oxford, Ohio 45056, USA; 8 Department of Biological Sciences, University of Alaska-Anchorage, Anchorage, Alaska 99508, USA; 9 School of Life Sciences, Sun Yat-Sen University, Guangzhou 510275, China; 10 Lawrence Berkeley National Laboratory, Berkeley, California 94720, USA

## Abstract

Analysis of the increasing wealth of metagenomic data collected from diverse environments can lead to the discovery of novel branches on the tree of life. Here we analyse 5.2 Tb of metagenomic data collected globally to discover a novel bacterial phylum (‘*Candidatus* Kryptonia') found exclusively in high-temperature pH-neutral geothermal springs. This lineage had remained hidden as a taxonomic ‘blind spot' because of mismatches in the primers commonly used for ribosomal gene surveys. Genome reconstruction from metagenomic data combined with single-cell genomics results in several high-quality genomes representing four genera from the new phylum. Metabolic reconstruction indicates a heterotrophic lifestyle with conspicuous nutritional deficiencies, suggesting the need for metabolic complementarity with other microbes. Co-occurrence patterns identifies a number of putative partners, including an uncultured *Armatimonadetes* lineage. The discovery of Kryptonia within previously studied geothermal springs underscores the importance of globally sampled metagenomic data in detection of microbial novelty, and highlights the extraordinary diversity of microbial life still awaiting discovery.

## Abstract (web summary)

The analysis of existing metagenomic data can lead to discovery of new microorganisms. Here, Eloe-Fadrosh *et al*. perform a large-scale analysis of global metagenomic data, followed by genome reconstruction and single-cell genomics, to describe a new bacterial phylum that inhabits geothermal springs.

## Graphical abstract

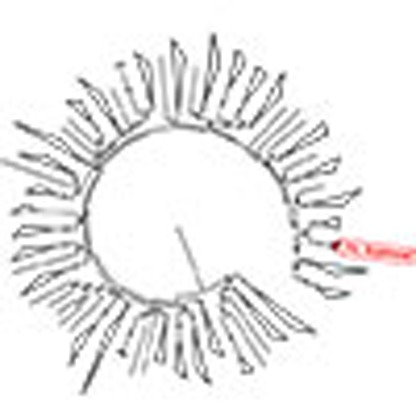

Molecular environmental surveys have provided a sizeable snapshot of microbial phylogenetic diversity. Sequencing of small-subunit ribosomal RNA (SSU rRNA) genes directly from the environment has expanded the known microbial tree of life from Woese's original 12 phyla to more than 70 bacterial phyla^1^,^2^. Advances in cultivation-independent methods for examining uncultured microbes, including single-cell genomics and deep sequencing of environmental samples, have begun yielding complete or near-complete genomes from many novel lineages^3^,^4^,^5^,^6^,^7^,^8^,^9^,^10^. These approaches have already led to the recovery of genomic information from a wealth of candidate lineages (phylogenetic lineages for which a cultured representative is not available), notably the Lokiarchaeota^11^, Pacearchaeota and Woesearchaeota^10^, and members of the Candidate Phyla Radiation^3^. These lineages, previously recognized only through SSU rRNA data and residing in poorly sampled habitats, are providing a more complete topology of the tree of life.

More recently, it has been suggested that a wealth of novel bacterial and archaeal clades exist that are systematically under-represented (the ‘rare biosphere') or missed altogether in classical surveys, leaving significant taxonomic ‘blind spots'^12^. Compared with many of the proposed candidate phyla for which SSU rRNA gene information exists, these taxonomic ‘blind spots' are uncharted lineages with potentially important ecological and evolutionary implications. Further, these lineages may be highly abundant and hold important metabolic or functional roles within the community, yet have been overlooked thus far in ecological surveys. Metagenome sequencing is uniquely suited for uncovering taxonomic ‘blind spots' since it does not suffer from biases introduced during PCR amplification, and has limitations only with insufficient resolution of minor populations within a community. However, an exploration of the complete compendium of available metagenomic sequences for the presence of taxonomic ‘blind spots' has yet to be performed^13^. Here, we report the results of large-scale mining of metagenomic data and single-cell genomics, which led to the discovery of a new bacterial phylum in geographically distinct geothermal springs.

## Results

### Identification of a novel bacterial candidate phylum

**Figure 1:**
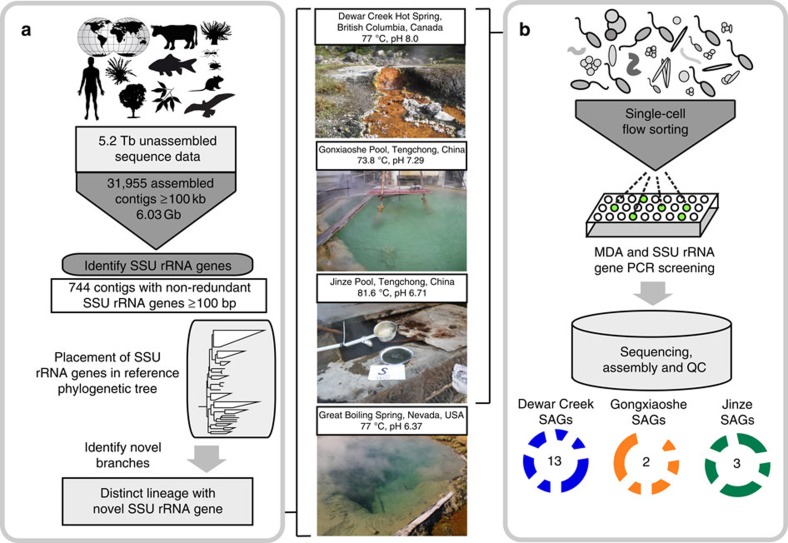
New lineage identified using metagenomic and single-cell genomic approaches. Workflow used to (**a**) identify novel SSU rRNA gene sequences globally, along with (**b**) single-cell genomics pipeline to screen and sequence single cells isolated from geothermal springs samples. For the three geothermal spring environments, we sequenced 13, 2 and 3 SAGs, respectively. SSU rRNA gene, small-subunit ribosomal gene; MDA, multiple displacement amplification; QC, quality control; SAG, single-amplified genome. The photograph of Jinze Pool, Tengchong, China, was taken from ref. 22.

To cast a global net for the discovery of novel microbial lineages in the absence of biases introduced via PCR amplicon-based surveys, we collected long assembled contigs (⩾100 kbp) from a comprehensive collection of 4,290 metagenomic data sets available through the Integrated Microbial Genomes with Microbiome Samples (IMG/M), a database containing a total of more than 5 Tb of sequence data^14^. From these data, 31,955 assembled contigs were identified and 744 contigs were further selected that contained SSU rRNA gene fragments >100 bp (Fig. 1a). The SSU rRNA gene sequences were then aligned and phylogenetically placed on a reference tree consisting of high-quality SSU rRNA sequences from bacteria and archaea^15^,^16^. Exploration of the constructed SSU rRNA tree for novel phylogenetic branches led to the identification of a distinct lineage consisting of a full-length SSU rRNA sequence. A subsequent search against all assembled metagenomic data identified three additional full-length SSU rRNA sequences. The four SSU rRNA gene sequences were from four geographically distant, high-temperature, pH-neutral, geothermal springs in North America and Asia (Fig. 1). These sequences shared an average 97.4% identity (±1.97% s.d.), and showed a maximum identity of only 83% to SSU rRNA genes (such as the one in GenBank ID: AP011715) in NCBI's Non-Redundant (NR) database. In line with the notion of taxonomic ‘blind spots'^12^, a comparison of ‘universal' SSU rRNA primer sets typically used for full-length and hypervariable region amplification with the four novel sequences indicated numerous mismatches, explaining why members of this lineage likely eluded detection in previous microbial diversity surveys (Supplementary Fig. 1; Supplementary Table 1).

Phylogenetic analysis of the four SSU rRNA genes placed the newly discovered lineage into a monophyletic branch within the *Fibrobacteres-Chlorobi-Bacteroidetes* (FCB) superphylum^9^,^17^ (Supplementary Fig. 2). Based on suggested thresholds for SSU rRNA sequence identity to distinguish new phyla^2^,^18^, we propose that this lineage represents a new bacterial candidate phylum (Supplementary Table 2).

### Comparative genomics and cell morphology.

**Figure 2:**
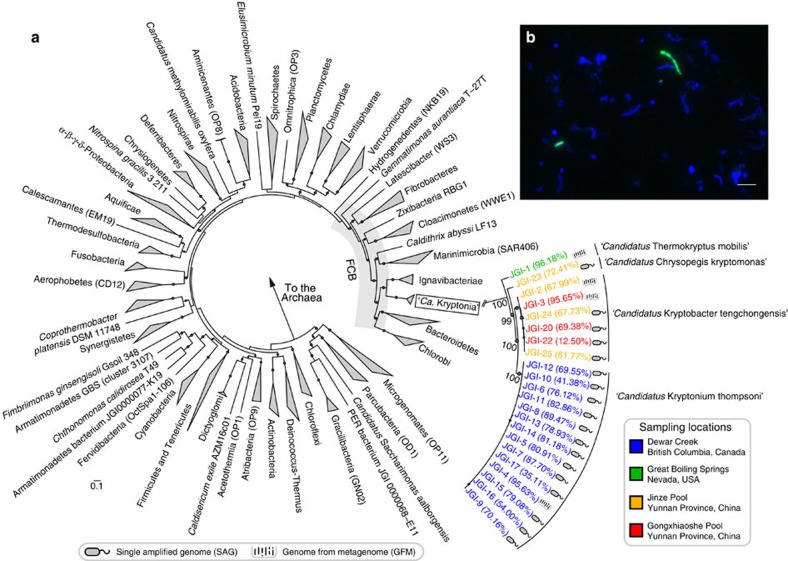
Maximum likelihood concatenated protein phylogeny and cell imaging for ‘*Ca.* Kryptonia.' (**a**) Phylogeny was based on concatenation of 56 conserved marker proteins, where at least 10 marker proteins were used to infer SAG phylogenetic placement (with the exception of JGI-22 with only six marker proteins recovered). Bootstrap support values ⩾50% are shown with small circles on nodes with robust phylogenetic support. The FCB superphylum is shown in the grey shaded region. Expanded phylogenetic tree for ‘*Ca.* Kryptonia' shows the placement of the proposed four genera represented by GFMs and SAGs, along with the estimated genome completeness shown in parentheses. (**b**) A ‘*Ca.* Kryptonia'-specific FISH (fluorescence *in situ* hybridization) probe was designed and used to visualize cells from Dewar Creek Spring sediment samples. ‘*Ca.* Kryptonia' cells hybridizing with the probe are green, while other cells are visualized with 4',6-diamidino-2-phenylindole (DAPI; blue). Scale bar, 5 μm.

Reassembly of the metagenomic data combined with tetranucleotide-based binning methods using the initial contigs containing the SSU rRNA genes yielded near-complete recovery of four distinct genomes, each from one of the four spring samples (Supplementary Fig. 3; Supplementary Table 3). Phylogenetic analysis of conserved marker genes supported its placement as a sister phylum to the *Ignavibacteria* with 100% bootstrap support (Fig. 2a; Supplementary Fig. 4). Three of the genomes reconstructed from metagenomes (GFMs) from Dewar Creek Spring, Canada^19^, Great Boiling Spring, Nevada^20^,^21^ and Gongxiaoshe pool, Yunnan Province, China^22^ had an average 95.8% estimated coverage, while the genome from Jinze pool, Yunnan Province, China^22^ had a lower estimated coverage of 68% (Supplementary Table 4). The high genomic sequence coverage across the four metagenomes (average 31.2 × coverage; Supplementary Table 3) suggested that this novel lineage might exist at sufficient cell abundance to be captured by single-cell technology. We therefore employed high-throughput single-cell isolation, whole-genome amplification (WGA) and SSU rRNA screening of single-amplified genomes (SAGs) in search for the novel lineage (Fig. 1). We successfully recovered a total of 18 SAGs from three of the four samples, corresponding to the novel phylum-level clade with an estimated average genome completeness of 67.2% (±20.1 s.d.) (Supplementary Table 3). We designate this new candidate phylum ‘*Candidatus* Kryptonia,' from the Greek word ‘*krupton'* meaning hidden or secret since it has hitherto eluded detection due to SSU rRNA primer biases (Supplementary Table 4).

The average nucleotide identity (ANI) -based metric, Microbial Species Identifier (MiSI), was used to compare the four ‘*Ca.* Kryptonia' GFMs and the 18 SAGs (ref. 23). This analysis revealed that almost all of the genotypes extracted from the same sample belonged to a single species (Supplementary Data 1). For example, the GFM reconstructed from Dewar Creek (‘*Ca.* Kryptonium thompsoni' JGI-4) and the 13 SAGs (‘*Ca.* Kryptonium thompsoni' JGI-5—JGI-17) collected from the same site shared an ANI of 99.67% (±0.15 s.d.) and represent a single coherent species^23^. A single exception to the above observations was the recovery of a divergent ‘*Ca.* Kryptonia' SAG (‘*Ca.* Chrysopegis kryptomonas' JGI-23) from the Jinze pool, Yunnan Province, China representing a population distinct from the other two SAGs recovered from this site (‘*Ca.* Kryptobacter tengchongensis' JGI-24 and JGI-25) (Supplementary Data 1). Across the four geothermal springs, the GFMs and SAGs collectively share average ANIs of only 78.86% (±1.42 s.d.), suggesting that they represent different genera of ‘*Ca.* Kryptonia'. Further support for genus-level designations is evident from nuanced functional and metabolic differences across the genomes, as described below.

In addition to recovering single cells of ‘*Ca.* Kryptonia' for genome amplification, we designed a SSU rRNA-targeted fluorescence *in situ* hybridization (FISH) probe to visualize cell morphology (Fig. 2b). The targeted ‘*Ca.* Kryptonia' cells appeared filamentous, and exhibited morphological heterogeneity ranging from short to elongated filaments. These findings are consistent with numerous reports describing filamentous thermophilic bacteria, most notably cultivated members of the sister phylum *Ignavibacteria* that range in length from 1 μm to >15 μm (refs 24, 25).

### CRISPR-Cas fusion and limited biogeographic distribution

CRISPR (clustered regularly interspaced short palindromic repeats) elements and *cas* (CRISPR-associated) genes across the ‘*Ca.* Kryptonia' genomes were recovered, and are suggestive of defense against viral attack. A novel fusion between two different CRISPR-Cas types (types I and III; subtypes I-B and III-A) was identified in all genomes. This unusual fusion contained the full gene set for components responsible for the multistep CRISPR processes for spacer acquisition, CRISPR locus transcription and maturation, and final nucleic acid interference^26^,^27^ (Supplementary Fig. 5). This observation represents the first report of a type I-B/type III-A CRISPR-Cas fusion and expands the known genetic diversity of CRISPR-Cas loci. Based on reconstruction of repeat-spacer arrays, the ‘*Ca.* Kryptonium thompsoni' genomes appear to represent a clonal CRISPR population without active spacer acquisition, while the ‘*Ca.* Kryptobacter tengchongensis' genomes are considerably dynamic in terms of a mosaic spacer collection (Supplementary Note 1; Supplementary Data 2 and 3). These findings suggest that the CRISPR-Cas encoded by ‘*Ca.* Kryptobacter tengchongensis' is highly active, while the ‘*Ca.* Kryptonium thompsoni' genomes are not actively acquiring spacers through the CRISPR-Cas system.

**Figure 3:**
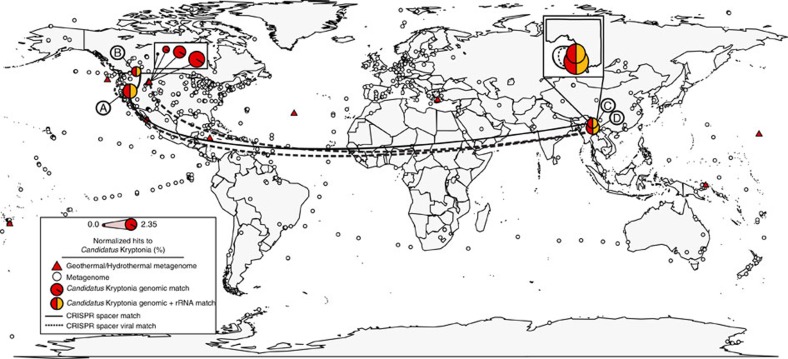
Limited, yet widely dispersed biogeographic distribution of ‘*Ca.* Kryptonia' genomes and CRISPR spacers. All genomic content from the ‘*Ca.* Kryptonia' GFMs and SAGs was used to comprehensively search the collection of 640 Gb of assembled metagenomic data from 4,290 environmental samples, including 169 samples from geothermal springs and hydrothermal vents denoted by red triangles (temperature ⩾50 °C). Marked circles are as follows: (A) Great Boiling Spring, Nevada^20^,^21^; (B) Dewar Creek Spring, Canada^19^; (C) Jinze pool, Yunnan Province, China^22^; and (D) Gongxiaoshe pool, Yunnan Province, China^22^. Significant matches were determined for sequences ⩾250 bp in length and with ⩾75% identity threshold for non-ribosomal genomic regions. For metagenomic contigs mapping to the ‘*Ca.* Kryptonia' ribosomal operon, a 97% identity threshold was used to capture only high-quality matches to ‘*Ca.* Kryptonia.' For CRISPR spacers, only significant matches allowing for up to 3 bp mismatch along the entire length of the spacer were considered. The ‘*Ca.* Kryptonia' genomic hits can be found in Supplementary Data 4 and the manually curated spacer hits can be found in Supplementary Data 3.

To verify the limited biogeographic distribution of ‘*Ca.* Kryptonia,' we systematically surveyed the collection of 640 Gb of assembled metagenomic data from 4,290 environmental samples (including 169 samples from geothermal springs and hydrothermal vents) for the presence of a genomic signature beyond our initial search using SSU rRNA fragments from 100 kbp contigs (Fig. 3; Supplementary Data 4). Further, we searched against all available SSU rRNA data from the SILVA database (ref. 16) for additional ‘*Ca.* Kryptonia' phylotypes and did not recover a highly similar match. Using this expanded search, we found evidence for ‘*Ca.* Kryptonia' in a total of 20 metagenomes, which included only three additional geographic sites compared to our initial SSU rRNA survey (Supplementary Data 4). The environments where this phylum was found were similar to the settings where we first discovered the genomic presence of ‘*Ca.* Kryptonia': all were high-temperature (⩾70 °C), pH-neutral (6.4–8.0) settings. In sum, the limited range of ‘*Ca.* Kryptonia' is reflected in the observation that genomic signatures were found in nine unique geographical locations from a total of 23 pH-neutral hot springs currently sampled by metagenomics, and absent from the 1,614 unique locations represented by 4,290 metagenomic samples.

In addition metagenomic searches specific for all CRISPR repeat-spacer arrays collected from the ‘*Ca.* Kryptonia' genomes resulted in a similar pattern of limited biogeographic distribution (Fig. 3; Supplementary Data 3). We identified shared spacers across ‘*Ca.* Kryptonia' populations in geographically distinct geothermal springs. For example, shared spacers were identified between the ‘*Ca.* Kryptobacter tengchongensis' JGI-2 and JGI-3 genomes despite sampling from separate geothermal pools in China. Further, shared spacers were identified across exceptionally wide geographic distances including Canada and Nevada (‘*Ca.* Kryptonium thompsoni' JGI-4 and the Great Boiling Springs metagenome), and China and Nevada (‘*Ca.* Kryptobacter tengchongensis' JGI-2 and the Great Boiling Springs metagenome) (Fig. 3). Remarkably, we also found spacer matches to a set of metagenomic contigs that we assigned as viral because of their linkage to known viral genes, from these same samples and metagenome samples collected from Yellowstone National Park^28^ (Fig. 3; Supplementary Note 1, Supplementary Fig. 5 and Supplementary Data 5). These genomic recruitment and spacer signature data suggest that ‘*Ca.* Kryptonia' is present in additional geothermal spring sites and that viruses which appear to infect ‘*Ca.* Kryptonia' circulate across wide geographic space as revealed from the conserved infection vestiges.

### Metabolic potential of ‘*Candidatus* Kryptonia'

The availability of multiple nearly complete ‘*Ca.* Kryptonia' genomes from both GFMs and SAGs enabled metabolic and putative functional predictions for this novel candidate phylum, as well as insights into some of the unique properties and notable absence of function for the individual genera. Approximately 50% of the predicted composite proteome for the ‘*Ca.* Kryptonia' genomes showed similarity to a diverse array of FCB superphylum members, with 11.3% and 1.96% best matches to thermophilic members of the phylum *Ignavibacteria* and *Caldithrix abyssi*, respectively (Supplementary Fig. 6). The conserved Por secretion system C-terminal sorting domain (TIGR04183), found exclusively in members of the FCB superphylum^9^, was recovered in all GFMs and SAGs, and altogether totalled 811 predicted proteins across the ‘*Ca.* Kryptonia' genomes. Reverse gyrase, the presumptive gene indicator for the extreme thermophilic and hyperthermophilic lifestyle in bacteria and archaea^29^, was found in all ‘*Ca.* Kryptonia' genomes, which suggests that most, if not all members, of this lineage are extreme thermophiles or hyperthermophiles. Further, we found evidence for horizontal gene transfer of the reverse gyrase from the crenarchaeal order *Thermoproteales* (Supplementary Note 2; Supplementary Fig. 7) and hypothesize that ‘*Ca.* Kryptonia's' thermophilic traits might have been acquired via lateral gene transfer rather than ancestral inheritance.

**Figure 4:**
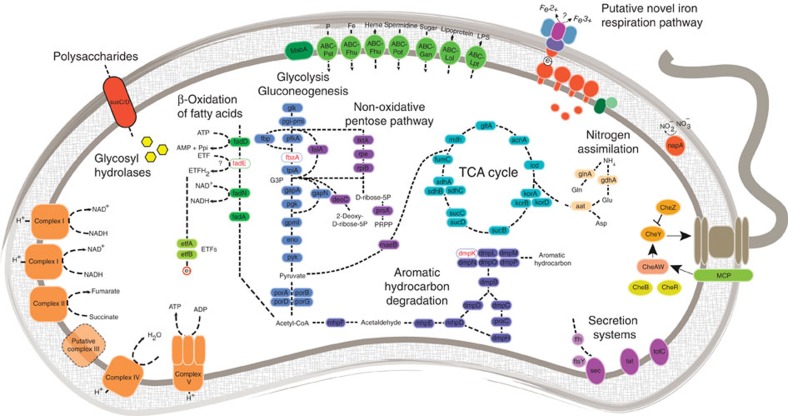
Reconstructed metabolic capacity of ‘*Ca.* Kryptonia.' Key metabolic predictions and novel features identified in ‘*Ca.* Kryptonia' GFM and SAGs, with full gene information available in Supplementary Data 6.

*‘Ca.* Kryptonia' is a motile heterotroph with a complete tricarboxylic acid cycle and key metabolic enzymes for Embden–Meyerhof glycolysis and the pentose phosphate pathway. We found evidence for a complex oxidative phosphorylation pathway, which points towards aerobic respiration (Fig. 4; Supplementary Data 6). An elaborate and unique respiratory pathway for the redox transformation of iron is encoded in the ‘*Ca.* Kryptonia' genomes with similar, yet non-homologous components to the well-characterized Mtr-like respiratory pathway^30^ (Supplementary Fig. 8). Altogether, ‘*Ca.* Kryptonia' has the machinery to carry out ferric iron respiration under thermophilic conditions and likely vies with archaeal community members to impact metal biogeochemistry in these geothermal springs.

*‘Ca.* Kryptonia' hosts the genomic potential for aromatic hydrocarbon degradation via oxidation to catechol, and subsequent catechol meta-cleavage (Fig. 4). Further, the ‘*Ca.* Kryptonium thompsoni' genomes encode a putative gene complement for the anaerobic degradation of aromatic amino acids or similar compounds, notably represented by a phenylacetyl-CoA oxidoreductase homologous to the hyperthermophilic archaeon *Ferroglobus placidus*^31^. This feature appears to be the first example of an extremely thermophilic or hyperthermophilic bacterium with the presumptive capacity to completely mineralize aromatic compounds, and holds biotechnological potential as well as implications for carbon cycling within geothermal springs^32^.

### Unexpected metabolic deficiencies identified in ‘Ca. *Kryptonia*'

An unexpected observation was that all ‘*Ca.* Kryptonia' genomes had conspicuous nutritional deficiencies, displaying gene loss for many biosynthetic pathways, including thiamine, biotin and amino acids, such as the evolutionarily conserved histidine biosynthesis^33^ (Fig. 4; Supplementary Data 6). While obligately host-dependent microbes and some free-living organisms with reduced genomes are known to omit a suite of anabolic pathways^34^,^35^, the ‘*Ca.* Kryptonia' genomes do not appear to have signatures of either lifestyle. An analysis of 759 high-quality FCB superphylum genomes indicate the near-complete ‘*Ca.* Kryptonia' genomes are distinct from free-living microbes in terms of amino acid pathway coverage and genome size, yet are not highly reduced compared with obligate symbionts (Supplementary Fig. 9). These findings suggest that ‘*Ca.* Kryptonia' has potentially evolved functional dependency on other microbes to acquire necessary metabolic requirements.

**Figure 5:**
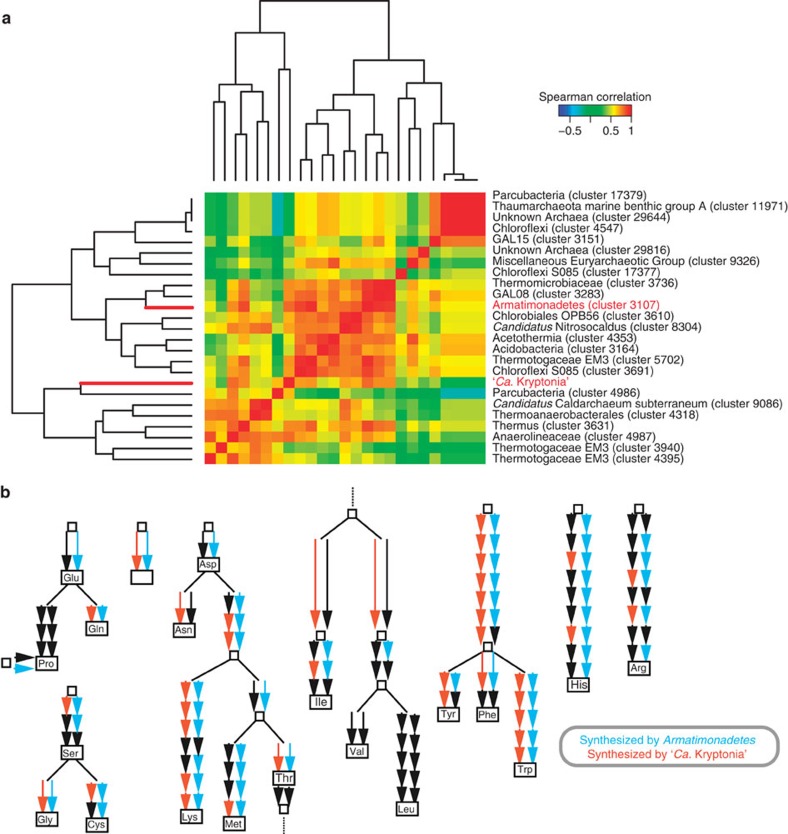
Co-occurrence patterns and metabolic complementarity with ‘*Ca.* Kryptonia.' (**a**) Spearman-rank correlation values were calculated based on reconstructed SSU rRNA sequences across 22 geothermal spring metagenomes, and led to the identification of a cluster of highly correlated phylotypes with ‘*Ca.* Kryptonia.' *Armatimonadetes* (cluster 3107) had the highest correlation value (*ρ*=0.82) with ‘*Ca.* Kryptonia.' (**b**) Biosynthetic pathways present in the *Armatimonadetes* genome which complement missing components in ‘*Ca.* Kryptonia.' Full gene information for the *Armatimonadetes* genome is available in Supplementary Data 7. Each arrow represents an enzymatic component of the biosynthetic pathways; arrows highlighted in blue are contributed by the *Armatimonadetes*, while arrows highlighted in dark orange are contributed by ‘*Ca.* Kryptonia.' Black arrows indicate enzyme was not recovered in either.

To explore the existence of possible microbial partners, we performed a co-occurrence analysis of SSU rRNA sequences retrieved through their targeted assembly from an expanded set of 22 geothermal springs metagenomes (Supplementary Note 3; Supplementary Table 5). An analysis of co-occurrence patterns for clusters of taxonomically coherent groups (clustered at 90% sequence identity) revealed a subset of taxonomically clustered groups (phylotypes) highly correlated with the abundance of ‘*Ca.* Kryptonia' (Supplementary Table 6). These clusters included an *Armatimonadetes* lineage, which had the highest correlation value, three separate lineages of *Chloroflexi*, and *Thermus* spp. (Fig. 5). For the twelve metagenomes in which ‘*Ca.* Kryptonia's' SSU rRNA was reconstructed, the *Armatimonadetes* lineage was found to co-occur in seven of those metagenomes at similar sequence coverage to the ‘*Ca.* Kryptonia' genomes, and was conspicuously absent across all other metagenomes surveyed. To explore the potential of the *Armatimonadetes* lineage to complement the metabolic deficiencies identified in ‘*Ca.* Kryptonia,' we reconstructed three nearly complete genomes of *Armatimonadetes* (Fig. 2; Supplementary Table 3; Supplementary Data 7) to infer metabolic potential and signatures of possible metabolic exchange and interaction. Analysis of the reconstructed genomes identified metabolic features complementary to those of ‘*Ca.* Kryptonia,' such as histidine, cysteine and methionine, proline, aspartic acid, and thiamine biosynthesis, and degradation of pentoses (Fig. 5b; Supplementary Note 4; Supplementary Data 7). Furthermore, in the reconstructed *Armatimonadetes* genomes we also identified a CsgG family protein, which forms transmembrane channels for secretion of ‘functional amyloids,' a class of bacterial proteins capable of assembling highly stable fibres through a nucleation–precipitation mechanism^36^. ‘Functional amyloids' play major roles in adhesion to surfaces and biofilm formation in diverse bacteria including *Escherichia coli*, *Caulobacter crescentus* and *Bacillus subtilis*^37^. Further, the CsgG-like transporter was located in a six-gene conserved cluster containing a predicted subtilase-family peptidase and a putative secreted protein with four copies of a ‘carboxypeptidase regulatory-like domain' (Pfam13620) (Supplementary Fig. 10). This domain is a member of the transthyretin clan and has been found to form amyloid in physiological conditions^38^. We hypothesize that this cluster in the *Armatimonadetes* genomes encodes for synthesis, secretion and assembly of ‘functional amyloid,' in which other members of the community may be embedded. On the other hand, the ‘*Ca.* Kryptonia' genomes encode many proteases and peptidases, which may be responsible for remodelling and digestion of this extracellular matrix.

Other co-occurring lineages with ‘*Ca.* Kryptonia' include the *Thermus* spp. cluster (Supplementary Table 6). Interestingly, ‘Ca. Kryptonia' might complement an incomplete denitrification pathway in *Thermus* spp., which may be responsible for high rates of nitrous oxide production^39^,^40^. *Thermus* spp. have been experimentally characterized to reduce nitrate to nitrous oxide but lack the capacity to subsequently produce dinitrogen^39^,^40^. ‘*Ca.* Kryptonia' encodes a nitrous oxide reductase (EC 1.7.2.4) but lacks other components of the denitrification pathway (Supplementary Note 5; Supplementary Table 7). Taken together, we hypothesize that ‘*Ca.* Kryptonia' may participate in a partnership with other organisms, such as the *Armatimonadetes*, or might interact with a broader consortium of microbes within the geothermal spring environment.

## Discussion

A comprehensive survey of a global set of assembled metagenomic data for novel microbial lineages has resulted in the discovery of a new bacterial candidate phylum in geothermal springs. The high-quality draft genome assemblies enabled by complementary approaches from metagenomic data and single-cell genomics data for ‘*Ca.* Kryptonia' faciliated delineation of the host–virus interaction across geographically distant sites. Further, we observed a novel fusion between two different CRISPR-Cas types, representing the first report of a type I-B/type III-A CRISPR-Cas fusion and expanded the known genetic diversity of CRISPR-Cas loci.

The metabolic capacity for ‘*Ca.* Kryptonia' provides evidence for a unique heterotrophic lifestyle with the putative capacity for iron respiration within a consistent ecological niche in geothermal springs. An unexpected observation was that all ‘*Ca.* Kryptonia' genomes had conspicuous nutritional deficiencies, which led to the hypothesis of a microbial partnership or interaction with a broader consortium of microbes. Subsequent genome reconstruction of genomes from a co-occurring *Armatimonadetes* lineage indicated potential complementarity for those metabolic features presumably absent in ‘*Ca.* Kryptonia.' It is well recognized that certain marine microbes, such as SAR11 (ref. 41) and SAR86 (ref. 42), lack a variety of anabolic pathways and likely rely on other microbial community members to supplement their requirements. Within geothermal springs, the growth of chlorophototroph *Ca.* Chloracidobacterium thermophilum in the laboratory was shown to depend upon two heterotrophs, *Anoxybacillus* and *Meiothermus* spp., because of the lack of biosynthetic pathways for branched-chain amino acids, lysine and cobalamin^43^. Our study suggests that dependency on other organisms within the geothermal spring community might be a more common occurrence than previously appreciated, perhaps contributing to challenges in obtaining many of these lineages as isolated monocultures. Future efforts to delineate this hypothesized interaction, particularly utilizing microscopy methods to visualize these uncultivated cells *in situ*, will further contribute to our understanding of ‘*Ca.* Kryptonia' and its role within the environment.

Geothermal springs have been heavily surveyed as a rich source of novel microbial branches on the tree of life^18^,^44^, yet our results indicate that additional phylogenetic novelty has yet to be captured from these environments. The discovery of a new candidate phylum emphasize that extraordinary microbial novelty is likely still awaiting discovery using the vast metagenomic data assembled from locations sampled globally.

## Methods

### Metagenomes

All publicly available metagenome data sets from IMG/M were used in the study (data accessed on 8 September 2014) (ref. 14). The metagenomes can be accessed at http://img.jgi.doe.gov and associated metadata can be found in the GOLD database at http://genomesonline.org.

### Metagenomic binning

Tetranucleotide-based binning methods were implemented as previously described to recover near-complete genomes from metagenomes^45^. Both single metagenomes and combined metagenome assemblies were used to recruit additional contigs that harboured the same tetranucleotide signature, and the raw reads were subsequently re-assembled using SPAdes version 3.1.0 (ref. 46).

### SAG generation

Sediment samples were collected from Dewar Creek hot spring (49.9543667°, −116.5155000°) near the source of the hot spring on 28 September 2012, from the Jinze pool (25.44138°, 98.46004°) on 12 August 2012, and from the Gongxiaoshe pool (25.44012°, 98.44081°) on 9 August 2011. Samples were mixed with 4% dimethylsulphoxide in TE buffer (1 mM EDTA, 10 mM Tris) for cryopreservation and stored at −80 °C within 24 hours of sample collection. Single cells were isolated using fluorescence-activated cell sorting, lysed and subjected to WGA as previously described^9^ with the following modifications: the alkaline lysis was preceded by a 20 min digest with lysozyme (Epicentre) at 30 °C; WGA was performed with a REPLI-g Single Cell Kit (Qiagen) with a scaled-down reaction volume of 2 μl; and the amplification reaction was incubated for 6 h at 30 °C. WGA reactions were diluted 10-fold, then aliquots were further diluted 200-fold for PCR screening targeting the V6–V8 regions (forward primer: 926wF (GAAACTYAAAKGAATTGRCGG ) and reverse primer: 1392R ( ACGGGCGGTGTGTRC )) of the SSU rRNA using a QuantiNova SYBR Green PCR kit (Qiagen) for 45 cycles of amplification^9^. PCR products were purified and sequenced, and SAGs matching ‘*Ca.* Kryptonia' SSU rRNA sequences were selected for shotgun sequencing.

### SAG sequencing, assembly and QC

Draft genomes for the eighteen SAGs were generated at the DOE Joint Genome Institute (JGI) using the Illumina MiSeq technology according to standard protocols (http://www.jgi.doe.gov/). Assembly was performed using SPAdes version 3.1.0 (ref. 46) using the -sc flag to denote MDA-derived data to account for uneven coverage of the single-cell genomes. Quality control and contaminant removal from the resultant assemblies was achieved using a two-step process. First, all assembled reads were used as input for a newly developed single-cell decontamination method (ProDeGe) (ref. 47), which uses both taxonomic and k-mer-based decisions to flag putative non-target contigs. Since the taxonomic information was limited to phylum-level designations, we further supplemented this procedure with direct mapping to the GFM data. For mapping, a combination of blast and blat were implemented to validate correct recruitment of the assembled SAG contigs to ‘*Ca.* Kryptonia'-specific GFM scaffolds. This method was important for retaining CRISPR/Cas genetic regions since ProDeGe had the tendency to flag these contigs based on divergent k-mer frequencies. Gene annotation was performed within the Integrated Microbial Genomes (IMG) platform developed by the DOE Joint Genome Institute^14^.

### SSU rRNA phylogeny

Full-length SSU rRNA gene sequences from ‘*Ca.* Kryptonia' were aligned using the SINA aligner (ref. 15) to a comprehensive database of references (SILVA-NR version 119) (ref. 16). A total of 187 full-length bacterial and archaeal reference sequences were selected based on taxonomic breadth from the SILVA database, and 1,354 distinct alignment patterns were used, and filtered using the *E. coli* positional mask. A maximum likelihood tree was calculated from the masked alignments with 100 bootstrap resamplings using the Generalized Time-Reversible model with G+I options in RAxML version 7.6.3 (raxmlHPC-PTHREADS-SSE3 -f a -x 12345 -p 12345 -# 100 -T 5 -m GTRGAMMAI) (ref. 48). To resolve placement within the FCB superphylum, a subset of 77 FCB superphylum members and 37 archaeal reference sequences were selected based on broad taxonomic representation within the FCB superphylum and phylogenies constructed using two separate algorithms with the GTR+G+I model: maximum likelihood (RAxML (ref. 48)) and Bayesian inference (MrBayes (ref. 49)). Node stability was evaluated using a rapid bootstrapping analysis (RAxML, 100 runs) and posterior probabilities (MrBayes, 2.4 million generations, burnin of 25%). Alignments and phylogenetic trees are available in Supplementary Data 8 and 9, respectively.

### Microscopy

An oligonucleotide probe specific for ‘*Ca.* Kryptonia' (Kryp56; 5′- CCGTGTCCCTGACTTGCA -3′) was designed in ARB (version 6.0.2) (ref. 50). The probe is a perfect match to 19 out of the 22 ‘*Ca.* Kryptonia' SSU rRNA gene sequences recovered in this study, and contains two or more mismatches to all SSU rRNA gene sequences in the SILVA-NR database (version 123) (ref. 16). The probe sequence was synthesized by Biomers.net (Ulm, Germany) with horseradish peroxidase conjugated to the 5' end. Cells from Dewar Creek sediment were separated from particulates by brief vortexing followed by centrifugation (30 s, 1,300*g*). Suspended cells were preserved with 4% dimethylsulphoxide and stored at −80 °C. The cells were permeabilized with lysozyme (10 mg ml^−1^ in TE buffer (1 mM EDTA, 10 mM Tris)) for 1 h at 37 °C and catalysed reporter deposition FISH (CARD-FISH) was performed based on the protocol of Pernthaler *et al*.^51^ Hybridization was carried out at 46 °C with 20% formamide, and the amplification was performed with tyramides conjugated to Alexa 488 (Life Technologies, #T20948). The optimal formamide concentration and specificity was predicted using mathFISH (ref. 52) and the DECIPHER ProbeMelt tool (ref. 53) (Supplementary Data 10), and confirmed empirically by performing CARD-FISH on the Dewar Creek cells over a gradient of formamide concentrations (10–35%). Samples were counterstained with 4',6-diamidino-2-phenylindole (DAPI) in VECTASHEILD Antifade Mounting Media (Vector Laboratories, #H-1200). Cells were visualized and imaged using a Leica DM6000B microscope using a HCX PL APO × 100 oil immersion objective.

### Conserved single-copy and housekeeping gene phylogenetic inference

A set of 56 universally conserved single-copy proteins in the Bacteria and Archaea was used for phylogenetic inference (Supplementary Data 11). Marker genes were detected and aligned with hmmsearch and hmmalign included in HMMER3 (ref. 54) using HMM profiles obtained from phylosift (http://phylosift.wordpress.com/)^55^. Alignments were concatenated and filtered^56^. Housekeeping genes were aligned using MAFFT with mafft-linsi option^57^. Best substitution model was selected using prottest^58^. Phylogeny was inferred using maximum likelihood methods with RAxML (version 7.6.3) (ref. 48). Tree topologies were tested for robustness using 100 bootstrap replicates with the LG+I+G model (raxmlHPC-PTHREADS-SSE3 -f a -x 12345 -p 12345 -# 100 -m PROTGAMMALG -T 5). Trees were visualized using Dendroscope^59^. The concatenated protein alignment and phylogenetic tree are available in Supplementary Data 12 and 13, respectively.

### Phylogenetic distribution of predicted proteins

The taxonomic distribution of all proteins across the GFM data along with the ‘*Ca.* Kryptonia' SAGs was compiled based on best matches to a comprehensive protein database of high-quality non-redundant bacterial and archaeal isolate genomes^14^. This search was performed using USEARCH (version 7.0) (ref. 60), where a protein match was considered for proteins with ⩾30% sequence identity across ⩾50% of the query alignment length. Phylogenetic affiliation at the phylum level was assigned for top matches, while proteins lacking a match according to the above criteria were noted as ‘no match.'

### Biogeography of ‘*Ca*. Kryptonia'

All genomic data for ‘*Ca.* Kryptonia' was searched against the assembled metagenomic data from 4,290 environmental samples using blat with the -fastMap option (ref. 61). Significant matches for non-ribosomal genomic regions were considered for sequences ⩾250 bp in length and with ⩾75% identity threshold. For metagenomic contigs mapping to the ribosomal operon, a 97% identity threshold was used to capture only high-quality matches to ‘*Ca.* Kryptonia.' Visualization of metagenomic matches globally was performed using the R package ‘maps' (ref. 62). All genomic matches can be found in Supplementary Data 4.

### CRISPR-Cas locus type determination

We used 99 CRISPR-associated (*cas*) gene sequence alignments and hidden Markov models from the TIGRFAM database (originally built by Haft *et al*.^63^ and later expanded by Zhang *et al*.^64^) to precisely find and identify Cas family members within the scaffolds of the ‘*Ca.* Kryptonia' genomes. We recovered and classified the corresponding CRISPR type for complete and partial CRISPR-Cas loci in all genomes following the unified CRISPR classification from 2011 (ref. 65).

### CRISPR repeat-spacer arrays analysis

The CRISPR Recognition Tool (CRT) (ref. 66) was used to detect CRISPR repeat-spacer regions across all ‘*Ca.* Kryptonia' assembled scaffolds using parameters according to the JGI's annotation pipeline^67^. In the case of ‘*Ca.* Thermokryptus mobilis' GFM JGI-1, we were unable to detect spacers, and therefore we additionally used the CRISPR assembler algorithm (Crass) (ref. 68) on the raw reads. Spacers were manually curated to cull false positives from the data set that clearly did not represent authentic spacer regions (in sum, 38 false positives). Potentially active repeat-spacer arrays were inferred based on direct association with a *cas* gene locus. We also considered the isolated repeat-spacers arrays when they shared the same repeat sequence with associated *cas* genes. CRISPRmap (refs 69, 70) was used to further characterize identified repeat regions. From a total of 1,031 trusted spacers, we next clustered these into 795 groups based on identity ⩾90% over the whole spacer length. Spacer groups were BLAST queried against distinct databases including ‘*Ca.* Kryptonia' genomes, reference public plasmid and viral data sets (from NCBI), and across the broad available metagenomic space (IMG/M).

### SSU rRNA gene assembly and co-occurrence analysis

Raw reads aligning to 16S and 18S rRNAs were collected for 22 metagenomes (Supplementary Table 5) from geothermal environments using hmmalign (ref. 54) against hmm models representing bacterial, archaeal and eukaryotic sequences^67^ and also by BBMap with default settings^71^ against sequences from the SILVA database (version 119) (ref. 16) dereplicated at 95% identity using UCLUST (ref. 60). Collected paired-end Illumina reads were merged using BBMerge (71) and assembled using Newbler (v. 2.8) (ref. 72) with -ml 60 -mi 99 -rip options. Resulting contigs and scaffolds were screened using cmalign from Infernal 1.1 package (ref. 73) and Rfam 16S and 18S rRNA models (RF00177.cm, RF01959.cm and RF01960.cm) (ref. 74). 16S and 18S rRNA sequences longer than 300 nt were retained and trimmed using cmalign against the best-matching model with the -matchonly option to remove introns. Reference sequences from the SILVA database were trimmed using cmalign with a domain-specific model and -matchonly option, and clustered together with 16S sequences extracted from shotgun metagenome data using UCLUST and percent identity cutoffs of 94, 92 and 90%. Clusters including sequences from at least two metagenome samples were retained and their abundances in metagenome samples were computed by multiplying the length of SSU rRNA sequence by the average coverage. Taxonomy was assigned to the clusters as last common ancestor of SILVA reference sequences included in the cluster, or as last common ancestor of SILVA sequences in the larger cluster obtained by co-clustering SILVA and metagenome sequences at 83% identity. Spearman's rank-order correlation of cluster abundances was used to estimate co-occurrence of the clusters in metagenome data.

## Additional information

**Accession codes:** Genome sequence data, assemblies and annotations have been deposited as Whole Genome Shotgun projects at DDBJ/EMBL/GenBank with the accession codes PRJEB11785 to PRJEB11788 (GFMs), and PRJEB11711 to PRJEB11728 (SAGs).

**How to cite this article:** Eloe-Fadrosh, E. A. *et al*. Global metagenomic survey reveals a new bacterial candidate phylum in geothermal springs. *Nat. Commun.* 7:10476 doi: 10.1038/ncomms10476 (2016).

## Supplementary Material

Supplementary Information Supplementary Figures 1-14, Supplementary Tables 1-7, Supplementary Notes 1- 5, and Supplementary References

Supplementary Data 1 Whole-genome based Average Nucleotide Identity (ANI) across all Ca. Kryptonia genotypes. Reconstructed genomes from metagenomes are designated as Genomes From Metagenomes (GFMs), along with sampling site. ANI values were calculated based on protein coding regions only, with tRNA and rRNA genes removed that would otherwise inflate the ANI according to Varghese et al., 2015.

Supplementary Data 2 Ca. Kryptonia spacer information. Manually curated table that includes: spacer_ID (identification number of Ca. Kryptonia genome spacers); spacer_group (identifier for those spacers that clustered together based on {greater than or equal to} 95% nt identity over the whole spacer length); type (CRISPR type of the spacer indicated, based on the information of the cas genes and/or the repeat sequence); columns JGI-1 - JGI-25 (indicate the spacer presence or absence across all Ca. Kryptonia genomes with a "x" or blank, respectively; proto-spacer (yes/no if there were spacer matches in putative viral contigs across all metagenomes compared against).

Supplementary Data 3 Ca. Kryptonia spacer hits (proto-spacers) across genomes and metagenomes. Manually curated table summarizing the distribution of Ca. Kryptonia spacers across genomes and metagenomes. BLAST results of Ca. Kryptonia spacers (minimum {greater than or equal to} 89% sequence identity over {greater than or equal to} 90% sequence length) against all metagenomic sequence space in the IMG/M database. For all the spacer groups derived from a CRISPR type I-B, we manually predicted the putative PAM (protospacer adjacent motif). %ID = percentage of identity; AL = alignment length; Ms = mismatches; Gap = gap openings; q.S = start coordinates of query; q.E = end coordinates of query; s.S = start coordinates of subject; s.E = end coordinates of subject; evalue = e-value; bitsc = bit score, q.L = query length; s.L = subject length; D = differential (q.L - AL). Blast hits and spacer queries are colored as follows: purple, Jinze; light green, Gongxiaoshe; dark green, Dewar Creek; and pink, GBS. Blast hits to Yellowstone National Park, GBS virome sample, and GBS spacers are marked in yellow, red, and orange, respectively.

Supplementary Data 4 Distribution of contigs across environmental metagenomes for Ca. Kryptonia. Total number of (A) Ca. Kryptonia non-rRNA and (B) rRNA-containing contigs. Contigs were identified from a search of 4,290 metagenomes with {greater than or equal to} 250 bp alignment to the genomic sequence. For rRNA matches, only sequences {greater than or equal to} 97% ID were considered.

Supplementary Data 5 Gene information for the scaffolds containing Ca. Kryptonia proto-spacers.

Supplementary Data 6 Predicted proteins in Ca. Kryptonia with functions in key metabolic pathways. KEGG ortholog ID, and module ID and name are shown. Isolate gene count represents the number of KEGG orthologs identified, while the genome count represents whether the ortholog was present or absent across the four GFMs and eighteen SAGs. KEGG orthologs highlighted in red indicate a putatively missing component from the pathway.

Supplementary Data 7 Predicted proteins in all Armatimonadetes genomes with functions in amino acid, thiamine, and biotin biosynthetic pathways. KEGG ortholog ID, and module ID and name are shown. Isolate gene count represents the number of KEGG orthologs identified, while the genome count represents whether the ortholog was present or absent across the three Armatimonadetes genomes. KEGG orthologs highlighted in red indicate a putatively missing component from the pathway.

Supplementary Data 8 SSU rRNA gene alignments.

Supplementary Data 9 SSU rRNA phylogenetic trees.

Supplementary Data 10 Prediction of optimal formamide concentration based on the DECIPHER ProbeMelt tool for the four Ca. Kryptonia SSU rRNA genes compared with all assembled SSU rRNA genes (350 sequences) from the four metagenomes. Region in gray (formamide concentrations 10-35%) were empirically confirmed by performing CARD-FISH on the Dewar Creek sample.

Supplementary Data 11 Conserved marker gene set used for phylogenetic tree construction and copy number identified across Ca. Kryptonia GFMs and SAGs.

Supplementary Data 12 Concatenated protein alignment.

Supplementary Data 13 Concatenated protein phylogenetic tree.

Supplementary Data 14 Predicted carbohydrate-active enzymes identified in Ca. Kryptonia. All predicted proteins were search against the CAzy (http://www.cazy.org/) database and only significant HMM matches with an e-value of {less than or equal to} 1e-5 were considered.
