# Supplementary material for: Global metagenomic survey reveals a new bacterial candidate phylum in geothermal springs
Source: Nat Commun. 2016 Jan 27;7:10476. doi: 10.1038/ncomms10476 (PMC4737851; doi:10.1038/ncomms10476)
Supplement: Supplementary Information — Supplementary Figures 1-14, Supplementary Tables 1-7, Supplementary Notes 1- 5, and Supplementary References [file ncomms10476-s1.pdf]

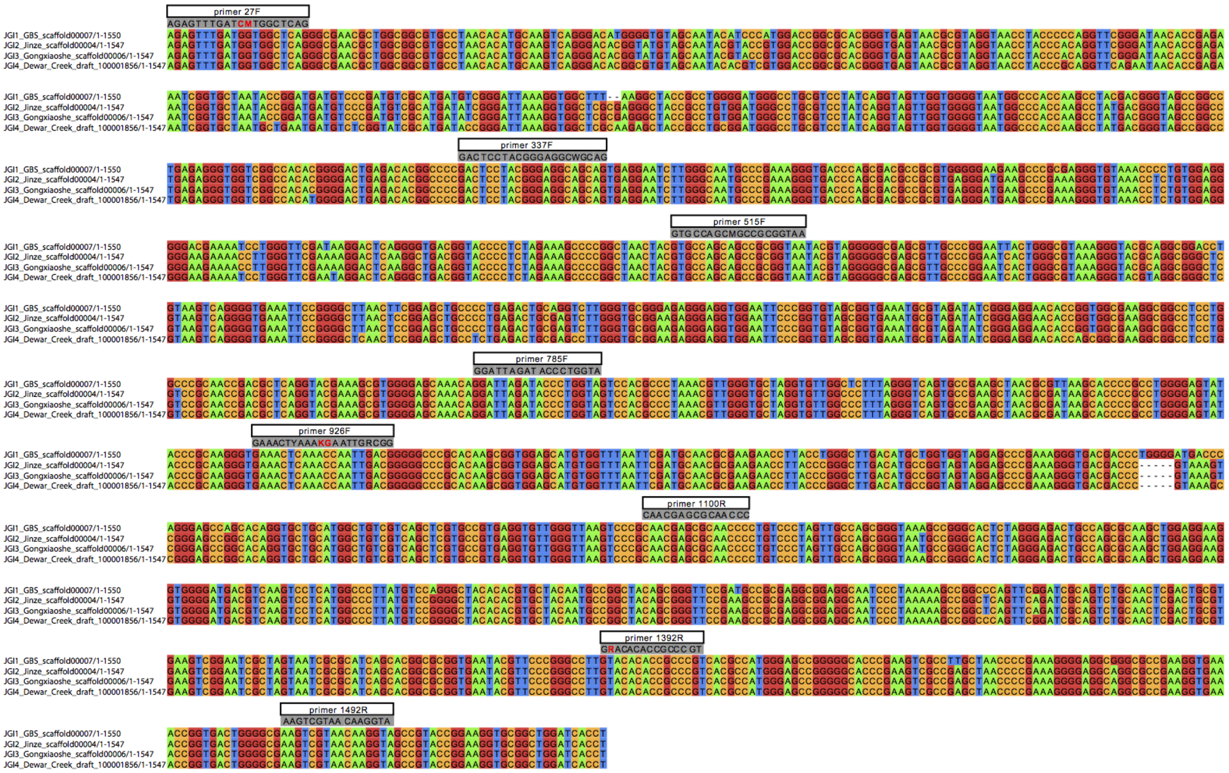

Supplementary Figure 1. Mismatches in SSU rRNA sequences with 'universal' bacterial primer sets. Alignment of four '*Ca.* Kryptonia' SSU rRNA gene sequences and relevant 'universal' bacterial primers showing mismatches in red. Primer sets used commonly in SSU rRNA surveys include: 27F (5′-AGAGTTTGATCMTGGCTCAG-3′), 337F (5′-GACTCCTACGGGAGGCWGCAG-3′), 515F (5′-GTGYCAGCMGCCGCGGTAA-3′), 785F (5′-GGATTAGATACCCTGGTA-3′), 926F (5′-GAAACTYAAAKGAATTGRCGG-3′), 1100R (5'-CAACGAGCGCAACCC-3'), 1392R (5′-GRACACACCGCCCGT-3′), and 1492R (5′-AAGTCGTAACAAGGTA-3′).

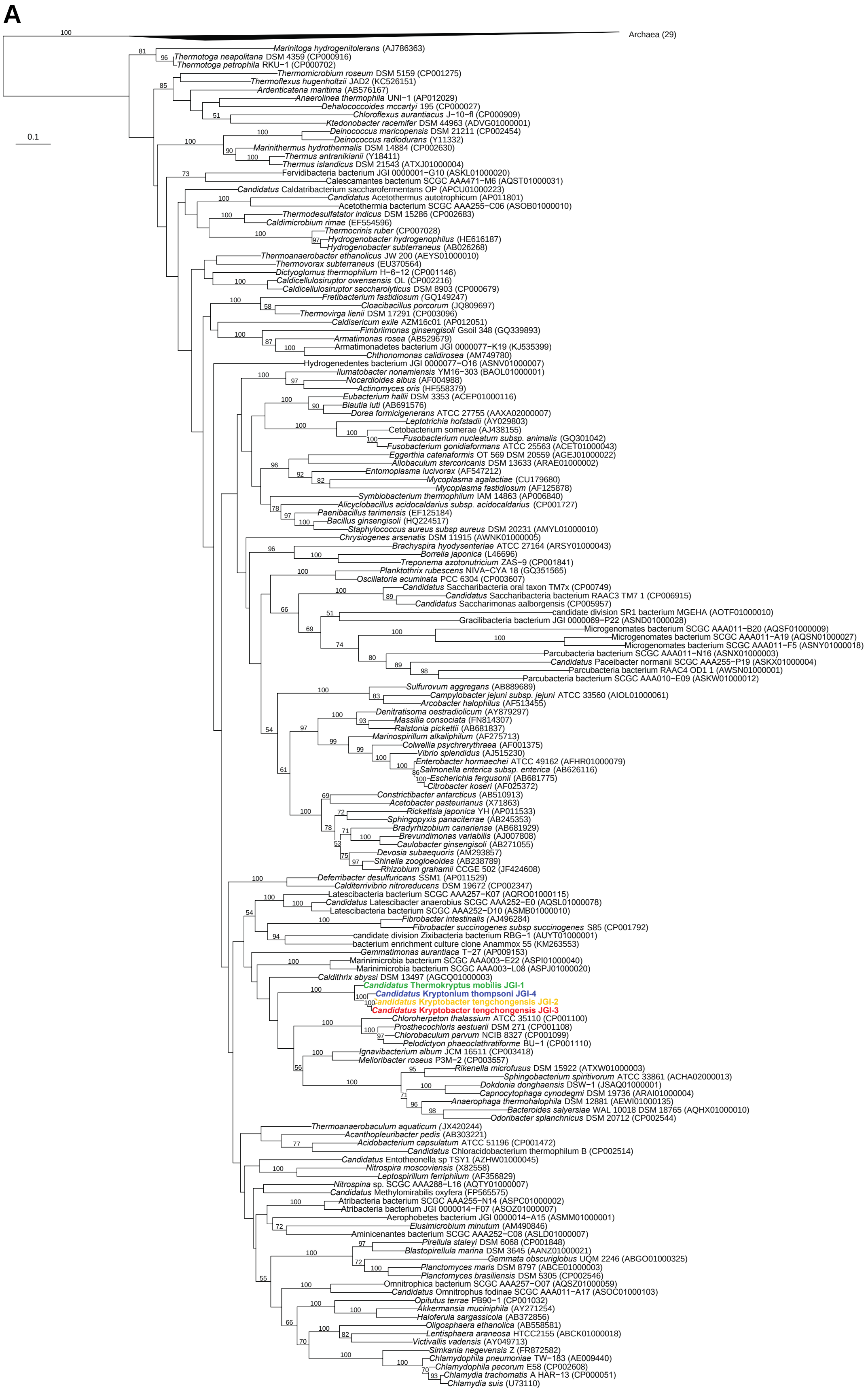
A
Archaea (29)
0.1
Marinitoga hydrogenitolerans (AJ786363)
Thermotoga neapolitana DSM 4359 (CP000916)
Thermotoga petrophila RKU−1 (CP000702)
Thermomicrobium roseum DSM 5159 (CP001275)
Thermoflexus hugenholtzii JAD2 (KC526151)
Ardenticatena maritima (AB576167)
Anaerolinea thermophila UNI−1 (AP012029)
Dehalococcoides mccartyi 195 (CP000027)
Chloroflexus aurantiacus J−10−fl (CP000909)
Ktedonobacter racemifer DSM 44963 (ADVG01000001)
Deinococcus maricopensis DSM 21211 (CP002454)
Deinococcus radiodurans (Y11332)
Marinithermus hydrothermalis DSM 14884 (CP002630)
Thermus antranikianii (Y18411)
Thermus islandicus DSM 21543 (ATXJ01000004)
Fervidibacteria bacterium JGI 0000001−G10 (ASKL01000020)
Calescamantes bacterium SCGC AAA471−M6 (AQST01000031)
Candidatus Caldatribacterium saccharofermentans OP (APCU01000223)
Candidatus Acetothermus autotrophicum (AP011801)
Acetothermia bacterium SCGC AAA255−C06 (ASOB01000010)
Thermodesulfatator indicus DSM 15286 (CP002683)
Caldimicrobium rimae (EF554596)
Thermocrinis ruber (CP007028)
Hydrogenobacter hydrogenophilus (HE616187)
Hydrogenobacter subterraneus (AB026268)
Thermoanaerobacter ethanolicus JW 200 (AEYS01000010)
Thermovorax subterraneus (EU370564)
Dictyoglomus thermophilum H−6−12 (CP001146)
Caldicellulosiruptor owensensis OL (CP002216)
Caldicellulosiruptor saccharolyticus DSM 8903 (CP000679)
Fretibacterium fastidiosum (GQ149247)
Cloacibacillus porcorum (JQ809697)
Thermovirga lienii DSM 17291 (CP003096)
Caldisericum exile AZM16c01 (AP012051)
Fimbriimonas ginsengisoli Gsoil 348 (GQ339893)
Armatimonas rosea (AB529679)
Armatimonadetes bacterium JGI 0000077−K19 (KJ535399)
Chthonomonas calidirosea (AM749780)
Hydrogenedentes bacterium JGI 0000077−O16 (ASNV01000007)
Ilumatobacter nonamiensis YM16−303 (BAOL01000001)
Nocardioides albus (AF004988)
Actinomyces oris (HF558379)
Eubacterium hallii DSM 3353 (ACEP01000116)
Blautia luti (AB691576)
Dorea formicigenerans ATCC 27755 (AAXA02000007)
Leptotrichia hofstadii (AY029803)
Cetobacterium somerae (AJ438155)
Fusobacterium nucleatum subsp. animalis (GQ301042)
Fusobacterium gonidiaformans ATCC 25563 (ACET01000043)
Eggerthia catenaformis OT 569 DSM 20559 (AGEJ01000022)
Allobaculum stercoricanis DSM 13633 (ARAE01000002)
Entomoplasma lucivorax (AF547212)
Mycoplasma agalactiae (CU179680)
Mycoplasma fastidiosum (AF125878)
Symbiobacterium thermophilum IAM 14863 (AP006840)
Alicyclobacillus acidocaldarius subsp. acidocaldarius (CP001727)
Paenibacillus tarimensis (EF125184)
Bacillus ginsengisoli (HQ224517)
Staphylococcus aureus subsp aureus DSM 20231 (AMYL01000010)
Chrysiogenes arsenatis DSM 11915 (AWNK01000005)
Brachyspira hyodysenteriae ATCC 27164 (ARSY01000043)
Borrelia japonica (L46696)
Treponema azotonutricium ZAS−9 (CP001841)
Planktothrix rubescens NIVA−CYA 18 (GQ351565)
Oscillatoria acuminata PCC 6304 (CP003607)
Candidatus Saccharibacteria oral taxon TM7x (CP00749)
Candidatus Saccharibacteria bacterium RAAC3 TM7 1 (CP006915)
Candidatus Saccharimonas aalborgensis (CP005957)
candidate division SR1 bacterium MGEHA (AOTF01000010)
Gracilibacteria bacterium JGI 0000069−P22 (ASND01000028)
Microgenomates bacterium SCGC AAA011−B20 (AQSF01000009)
Microgenomates bacterium SCGC AAA011−A19 (AQSN01000027)
Microgenomates bacterium SCGC AAA011−F5 (ASNY01000018)
Parcubacteria bacterium SCGC AAA011−N16 (ASNX01000003)
Candidatus Paceibacter normanii SCGC AAA255−P19 (ASKX01000004)
Parcubacteria bacterium RAAC4 OD1 1 (AWSN01000001)
Parcubacteria bacterium SCGC AAA010−E09 (ASKW01000012)
Sulfurovum aggregans (AB889689)
Campylobacter jejuni subsp. jejuni ATCC 33560 (AIOL01000061)
Arcobacter halophilus (AF513455)
Denitratisoma oestradiolicum (AY879297)
Massilia consociata (FN814307)
Ralstonia pickettii (AB681837)
Marinospirillum alkaliphilum (AF275713)
Colwellia psychrerythraea (AF001375)
Vibrio splendidus (AJ515230)
Enterobacter hormaechei ATCC 49162 (AFHR01000079)
Salmonella enterica subsp. enterica (AB626116)
Escherichia fergusonii (AB681775)
Citrobacter koseri (AF025372)
Constrictibacter antarcticus (AB510913)
Acetobacter pasteurianus (X71863)
Rickettsia japonica YH (AP011533)
Sphingopyxis panaciterrae (AB245353)
Bradyrhizobium canariense (AB681929)
Brevundimonas variabilis (AJ007808)
Caulobacter ginsengisoli (AB271055)
Devosia subaequoris (AM293857)
Shinella zoogloeoides (AB238789)
Rhizobium grahamii CCGE 502 (JF424608)
Deferribacter desulfuricans SSM1 (AP011529)
Calditerrivibrio nitroreducens DSM 19672 (CP002347)
Latescibacteria bacterium SCGC AAA257−K07 (AQRO01000115)
Candidatus Latescibacter anaerobius SCGC AAA252−E0 (AQSL01000078)
Latescibacteria bacterium SCGC AAA252−D10 (ASMB01000010)
Fibrobacter intestinalis (AJ496284)
Fibrobacter succinogenes subsp succinogenes S85 (CP001792)
candidate division Zixibacteria bacterium RBG−1 (AUYT01000001)
bacterium enrichment culture clone Anammox 55 (KM263553)
Gemmatimonas aurantiaca T−27 (AP009153)
Marinimicrobia bacterium SCGC AAA003−E22 (ASPI01000040)
Marinimicrobia bacterium SCGC AAA003−L08 (ASPJ01000020)
Caldithrix abyssi DSM 13497 (AGCQ01000003)
Candidatus Thermokryptus mobilis JGI-1
Candidatus Kryptonium thompsoni JGI-4
Candidatus Kryptobacter tengchongensis JGI-2
Candidatus Kryptobacter tengchongensis JGI-3
Chloroherpeton thalassium ATCC 35110 (CP001100)
Prosthecochloris aestuarii DSM 271 (CP001108)
Chlorobaculum parvum NCIB 8327 (CP001099)
Pelodictyon phaeoclathratiforme BU−1 (CP001110)
Ignavibacterium album JCM 16511 (CP003418)
Melioribacter roseus P3M−2 (CP003557)
Rikenella microfusus DSM 15922 (ATXW01000003)
Sphingobacterium spiritivorum ATCC 33861 (ACHA02000013)
Dokdonia donghaensis DSW−1 (JSAQ01000001)
Capnocytophaga cynodegmi DSM 19736 (ARAI01000004)
Anaerophaga thermohalophila DSM 12881 (AEWI01000135)
Bacteroides salyersiae WAL 10018 DSM 18765 (AQHX01000010)
Odoribacter splanchnicus DSM 20712 (CP002544)
Thermoanaerobaculum aquaticum (JX420244)
Acanthopleuribacter pedis (AB303221)
Acidobacterium capsulatum ATCC 51196 (CP001472)
Candidatus Chloracidobacterium thermophilum B (CP002514)
Candidatus Entotheonella sp TSY1 (AZHW01000045)
Nitrospira moscoviensis (X82558)
Leptospirillum ferriphilum (AF356829)
Nitrospina sp. SCGC AAA288−L16 (AQTY01000007)
Candidatus Methylomirabilis oxyfera (FP565575)
Atribacteria bacterium SCGC AAA255−N14 (ASPC01000002)
Atribacteria bacterium JGI 0000014−F07 (ASOZ01000007)
Aerophobetes bacterium JGI 0000014−A15 (ASMM01000001)
Elusimicrobium minutum (AM490846)
Aminicenantes bacterium SCGC AAA252−C08 (ASLD01000007)
Pirellula staleyi DSM 6068 (CP001848)
Blastopirellula marina DSM 3645 (AANZ01000021)
Gemmata obscuriglobus UQM 2246 (ABGO01000325)
Planctomyces maris DSM 8797 (ABCE01000003)
Planctomyces brasiliensis DSM 5305 (CP002546)
Omnitrophica bacterium SCGC AAA257−O07 (AQSZ01000059)
Candidatus Omnitrophus fodinae SCGC AAA011−A17 (ASOC01000103)
Opitutus terrae PB90−1 (CP001032)
Akkermansia muciniphila (AY271254)
Haloferula sargassicola (AB372856)
Oligosphaera ethanolica (AB558581)
Lentisphaera araneosa HTCC2155 (ABCK01000018)
Victivallis vadensis (AY049713)
Simkania negevensis Z (FR872582)
Chlamydophila pneumoniae TW−183 (AE009440)
Chlamydophila pecorum E58 (CP002608)
Chlamydia trachomatis A HAR−13 (CP000051)
Chlamydia suis (U73110)

B

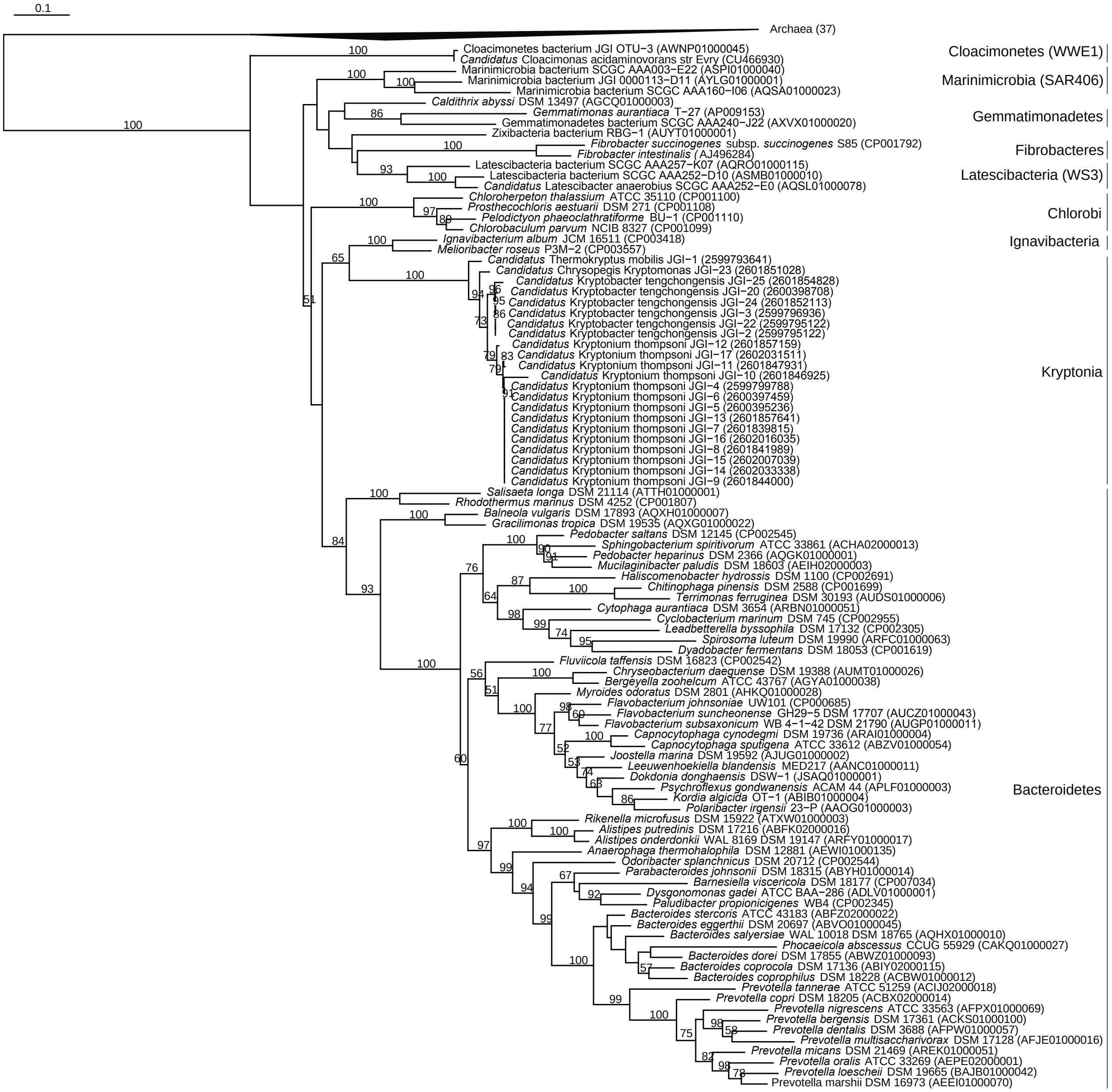
0.1
Archaea (37)
Cloacimonetes bacterium JGI OTU-3 (AWNP01000045)
Candidatus Cloacimonas acidaminovorans str Evry (CU466930)
Marinimicrobia bacterium SCGC AAA003-E22 (ASPI01000040)
Marinimicrobia bacterium JGI 0000113-D11 (AYLG01000001)
Marinimicrobia bacterium SCGC AAA160-I06 (AQSA01000023)
Caldithrix abyssi DSM 13497 (AGCQ01000003)
Gemmatimonas aurantiaca T-27 (AP009153)
Gemmatimonadetes bacterium SCGC AAA240-J22 (AXVX01000020)
Zixibacteria bacterium RBG-1 (AUYT01000001)
Fibrobacter succinogenes subsp. succinogenes S85 (CP001792)
Fibrobacter intestinalis (AJ496284)
Latescibacteria bacterium SCGC AAA257-K07 (AQRO01000115)
Latescibacteria bacterium SCGC AAA252-D10 (ASMB01000010)
Candidatus Latescibacter anaerobius SCGC AAA252-E0 (AQSL01000078)
Chloroherpeton thalassium ATCC 35110 (CP001100)
Prosthecochloris aestuarii DSM 271 (CP001108)
Pelodictyon phaeoclathratiforme BU-1 (CP001110)
Chlorobaculum parvum NCIB 8327 (CP001099)
Ignavibacterium album JCM 16511 (CP003418)
Melioribacter roseus P3M-2 (CP003557)
Candidatus Thermokryptus mobilis JGI-1 (2599793641)
Candidatus Chrysopegis Kryptomonas JGI-23 (2601851028)
Candidatus Kryptobacter tengchongensis JGI-25 (2601854828)
Candidatus Kryptobacter tengchongensis JGI-20 (2600398708)
Candidatus Kryptobacter tengchongensis JGI-24 (2601852113)
Candidatus Kryptobacter tengchongensis JGI-3 (2599796936)
Candidatus Kryptobacter tengchongensis JGI-22 (2599795122)
Candidatus Kryptobacter tengchongensis JGI-2 (2599795122)
Candidatus Kryptonium thompsoni JGI-12 (2601857159)
Candidatus Kryptonium thompsoni JGI-17 (2602031511)
Candidatus Kryptonium thompsoni JGI-11 (2601847931)
Candidatus Kryptonium thompsoni JGI-10 (2601846925)
Candidatus Kryptonium thompsoni JGI-4 (2599799788)
Candidatus Kryptonium thompsoni JGI-6 (2600397459)
Candidatus Kryptonium thompsoni JGI-5 (2600395236)
Candidatus Kryptonium thompsoni JGI-13 (2601857641)
Candidatus Kryptonium thompsoni JGI-7 (2601839815)
Candidatus Kryptonium thompsoni JGI-16 (2602016035)
Candidatus Kryptonium thompsoni JGI-8 (2601841989)
Candidatus Kryptonium thompsoni JGI-15 (2602007039)
Candidatus Kryptonium thompsoni JGI-14 (2602033338)
Candidatus Kryptonium thompsoni JGI-9 (2601844000)
Salisaeta longa DSM 21114 (ATTH01000001)
Rhodothermus marinus DSM 4252 (CP001807)
Balneola vulgaris DSM 17893 (AQXH01000007)
Gracilimonas tropica DSM 19535 (AQXG01000022)
Pedobacter saltans DSM 12145 (CP002545)
Sphingobacterium spiritivorum ATCC 33861 (ACHA02000013)
Pedobacter heparinus DSM 2366 (AQGK01000001)
Mucilaginibacter paludis DSM 18603 (AEIH02000003)
Haliscomenobacter hydrossis DSM 1100 (CP002691)
Chitinophaga pinensis DSM 2588 (CP001699)
Terrimonas ferruginea DSM 30193 (AUDS01000006)
Cytophaga aurantiaca DSM 3654 (ARBN01000051)
Cyclobacterium marinum DSM 745 (CP002955)
Leadbetterella byssophila DSM 17132 (CP002305)
Spirosoma luteum DSM 19990 (ARFC01000063)
Dyadobacter fermentans DSM 18053 (CP001619)
Fluviicola taffensis DSM 16823 (CP002542)
Chryseobacterium daeguense DSM 19388 (AUMT01000026)
Bergeyella zoohelcum ATCC 43767 (AGYA01000038)
Myroides odoratus DSM 2801 (AHKQ01000028)
Flavobacterium johnsoniae UW101 (CP000685)
Flavobacterium suncheonense GH29-5 DSM 17707 (AUCZ01000043)
Flavobacterium subsaxonicum WB 4-1-42 DSM 21790 (AUGP01000011)
Capnocytophaga cynodegmi DSM 19736 (ARAI01000004)
Capnocytophaga sputigena ATCC 33612 (ABZV01000054)
Joostella marina DSM 19592 (AJUG01000002)
Leeuwenhoekiella blandensis MED217 (AANC01000011)
Dokdonia donghaensis DSW-1 (JSAQ01000001)
Psychroflexus gondwanensis ACAM 44 (APLF01000003)
Kordia algicida OT-1 (ABIB01000004)
Polaribacter irgensii 23-P (AAOG01000003)
Rikenella microfusus DSM 15922 (ATXW01000003)
Alistipes putredinis DSM 17216 (ABFK02000016)
Alistipes onderdonkii WAL 8169 DSM 19147 (ARFY01000017)
Anaerophaga thermohalophila DSM 12881 (AEWI01000135)
Odoribacter splanchnicus DSM 20712 (CP002544)
Parabacteroides johnsonii DSM 18315 (ABYH01000014)
Barnesiella viscericola DSM 18177 (CP007034)
Dysgonomonas gadei ATCC BAA-286 (ADLV01000001)
Paludibacter propionicigenes WB4 (CP002345)
Bacteroides stercoris ATCC 43183 (ABFZ02000022)
Bacteroides eggerthii DSM 20697 (ABVO01000045)
Bacteroides salyersiae WAL 10018 DSM 18765 (AQHX01000010)
Phocaeicola abscessus CCUG 55929 (CAKQ01000027)
Bacteroides dorei DSM 17855 (ABWZ01000093)
Bacteroides coprocola DSM 17136 (ABIY02000115)
Bacteroides coprophilus DSM 18228 (ACBW01000012)
Prevotella tannerae ATCC 51259 (ACIJ02000018)
Prevotella copri DSM 18205 (ACBX02000014)
Prevotella nigrescens ATCC 33563 (AFPX01000069)
Prevotella bergensis DSM 17361 (ACKS01000100)
Prevotella dentalis DSM 3688 (AFPW01000057)
Prevotella multisaccharivorax DSM 17128 (AFJE01000016)
Prevotella micans DSM 21469 (AREK01000051)
Prevotella oralis ATCC 33269 (AEPE02000001)
Prevotella loescheii DSM 19665 (BAJB01000042)
Prevotella marshii DSM 16973 (AEEI01000070)
Cloacimonetes (WWE1)
Marinimicrobia (SAR406)
Gemmatimonadetes
Fibrobacteres
Latescibacteria (WS3)
Chlorobi
Ignavibacteria
Kryptonia
Bacteroidetes

C

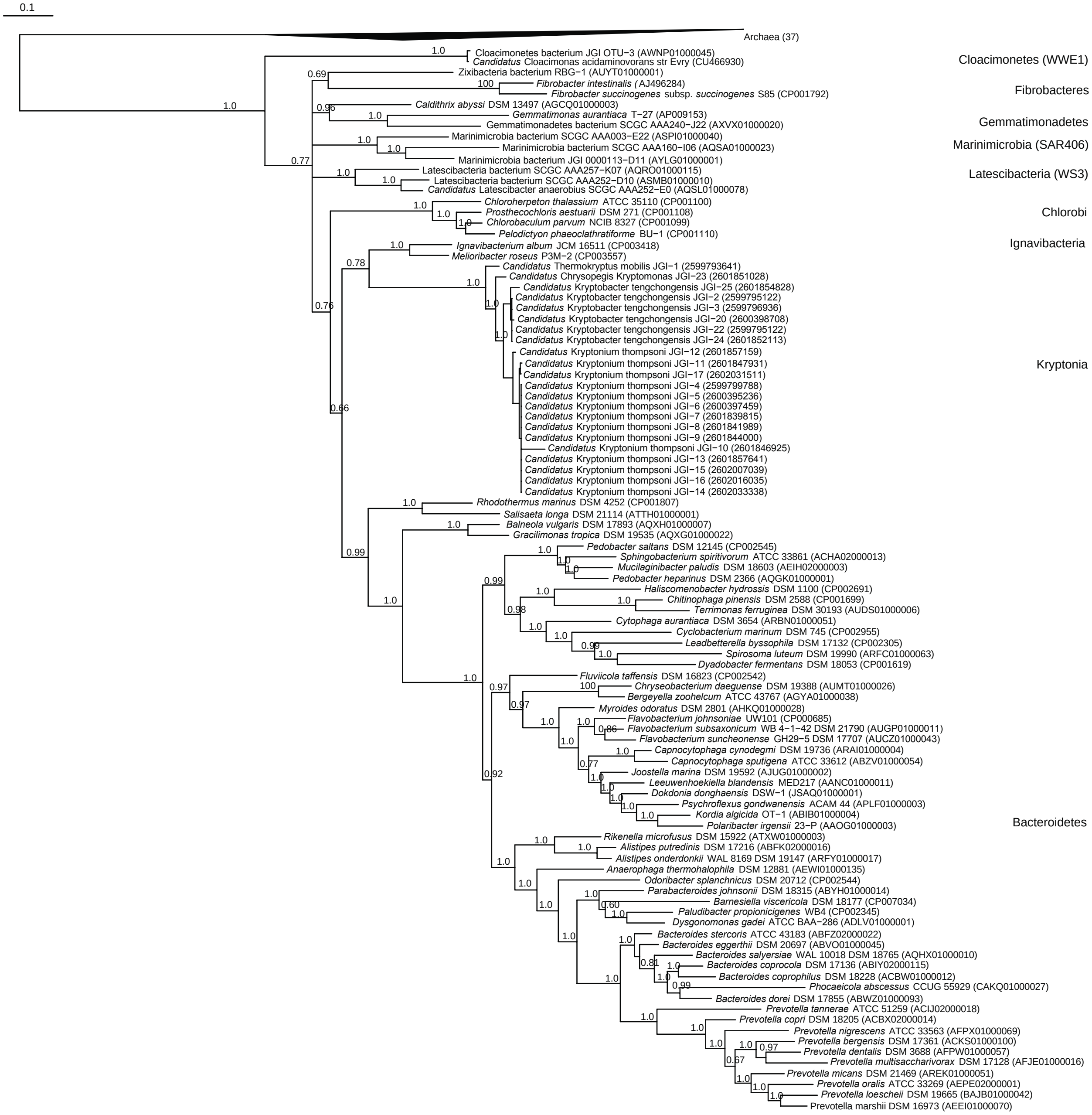

Supplementary Figure 2. SSU rRNA phylogeny. (A) Phylogenetic placement of the four full-length SSU rRNA genes from '*Ca.* Kryptonia' derived from the genomes reconstructed from metagenomic data. A total of 187 full-length bacterial and archaeal reference sequences were selected from the SILVA database, and 1,354 distinct alignment patterns were used. A maximum likelihood tree was calculated from the masked alignments with 100 bootstrap resamplings using RAxML under the Generalized Time-Reversible G+I model. Bootstrap support values ≥ 50% for all branches are shown. The four '*Ca.* Kryptonia' sequences are in bold, and are colored by their sampling locations (Green, Great Boiling Springs, Nevada, USA; Blue, Dewar Creek, British Columbia, Canada; Yellow, Jinze Pool, Yunnan province, China; and

Red, Gongxiaoshe Pool, Yunnan province, China). (**B**) Phylogenetic placement of all full-length '*Ca.* Kryptonia' SSU rRNA genes from the 4 genomes from metagenomes and the 18 single-cell genomes within the FCB superphylum. A total of 77 FCB superphylum members and 37 archaeal references sequences were selected, and 1,456 distinct alignment patterns were used. A maximum likelihood tree was calculated from the masked alignments with 100 bootstrap resamplings using RAxML under the Generalized Time-Reversible G+I model. Bootstrap support values ≥ 50% for all branches are shown. (**C**) Phylogenetic placement of all full-length '*Ca.* Kryptonia' SSU rRNA genes within the FCB superphylum using Bayesian inference (MrBayes). The same alignment from (**B**) was used with the GTR+G+I model to evaluate node stability with 2.4 million generations and a burnin of 25%. The posterior probability is for all nodes. For all phylogenies, NCBI accession numbers are shown in parentheses for references and IMG gene ids are shown in parentheses for '*Ca.* Kryptonia' SSU rRNA genes. All alignments and phylogenetic trees are available in Supplementary Data 8 and 9, respectively.

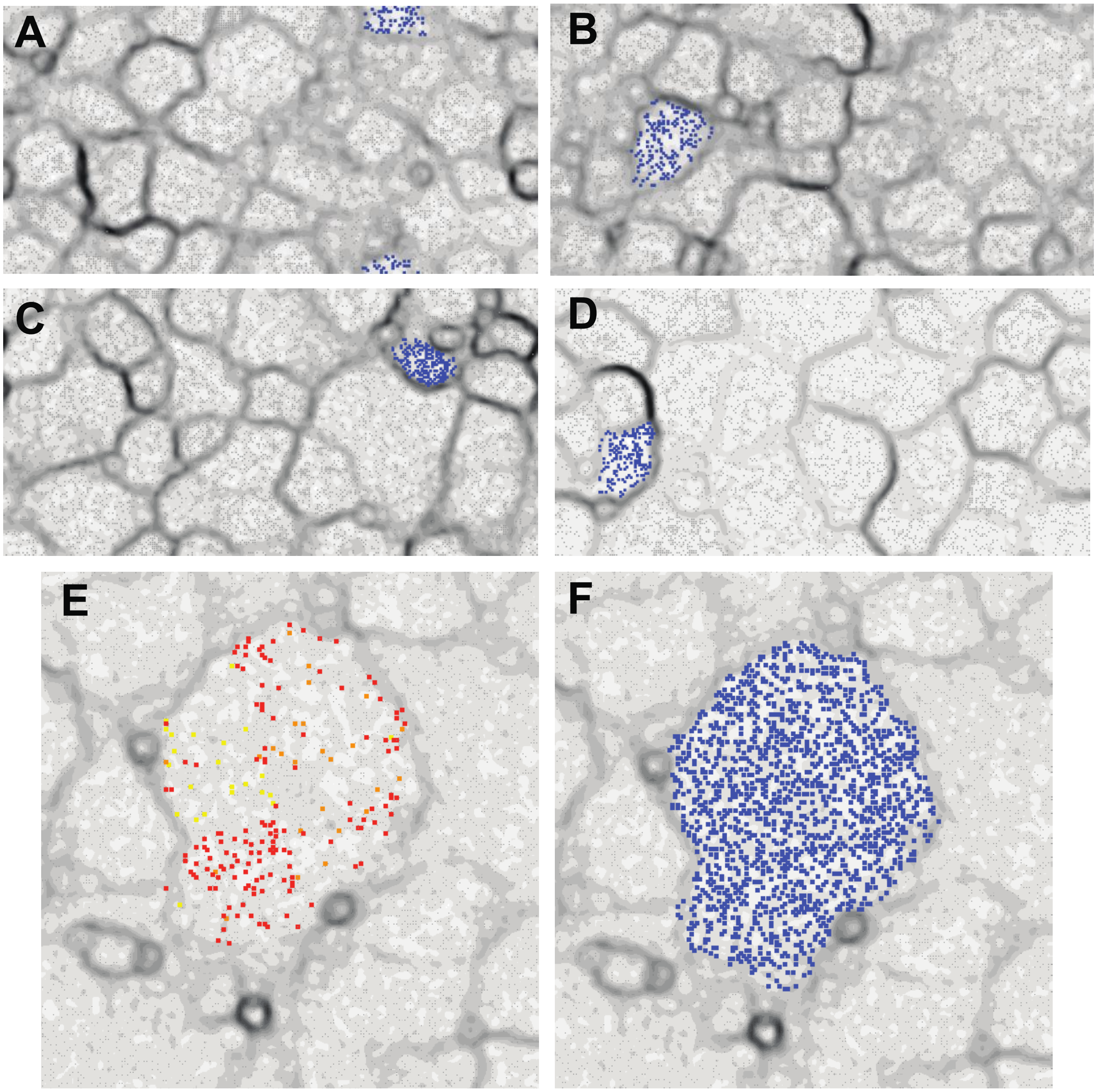

Supplementary Figure 3. Tetranucleotide frequency binning for ‘*Ca.* Kryptonia.’ Emergent self-organizing maps (ESOMs) were used for each metagenome and the combined metagenomes to identify genomic fragments affiliated with ‘*Ca.* Kryptonia’ (colored blue). **(A)** IMG metagenome ID 3300000083 (Great Boiling Spring, NV) with ‘*Ca.* Thermokryptus mobilis’ JGI-1 contigs highlighted. **(B)** IMG metagenome ID 3300000866 (Jinze, China) with ‘*Ca.* Kryptobacter tengchongensis’ JGI-2 contigs highlighted. **(C)** IMG metagenome ID 3300000865 (Gongxiaoshe, China) with ‘*Ca.* Kryptobacter tengchongensis’ JGI-3 contigs highlighted. **(D)** IMG metagenome ID 3300002851 (Dewar Creek, CA) with ‘*Ca.* Kryptonium thompsoni’ JGI-4 contigs highlighted. **(E)** Combined metagenomes identified as follows: ‘*Ca.* Thermokryptus mobilis’ JGI-1, yellow; ‘*Ca.* Kryptobacter tengchongensis’ JGI-3, orange; ‘*Ca.* Kryptonium thompsoni’ JGI-4, red. **(F)** Combined metagenomes with all recruited contigs affiliated with ‘*Ca.* Kryptonia.’

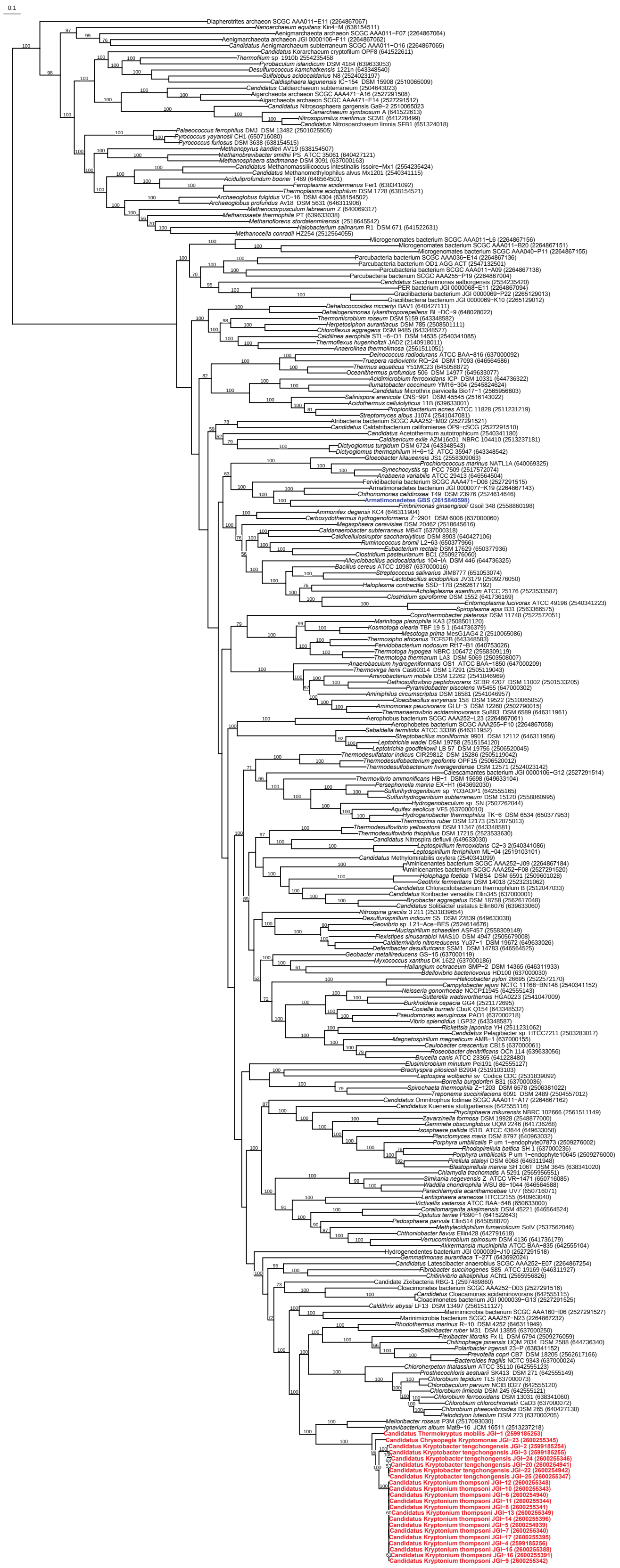

Supplementary Figure 4. Maximum likelihood concatenated protein phylogeny. Phylogeny based on 56 conserved marker proteins as shown in Fig. 2 with all genomes displayed with IMG genome ids in parentheses. All '*Ca.* Kryptonia' genomes (4 GFMs and 18 SAGs) are highlighted in red. The concatenated protein alignment and phylogenetic tree are available in Supplementary Data 12 and 13, respectively.

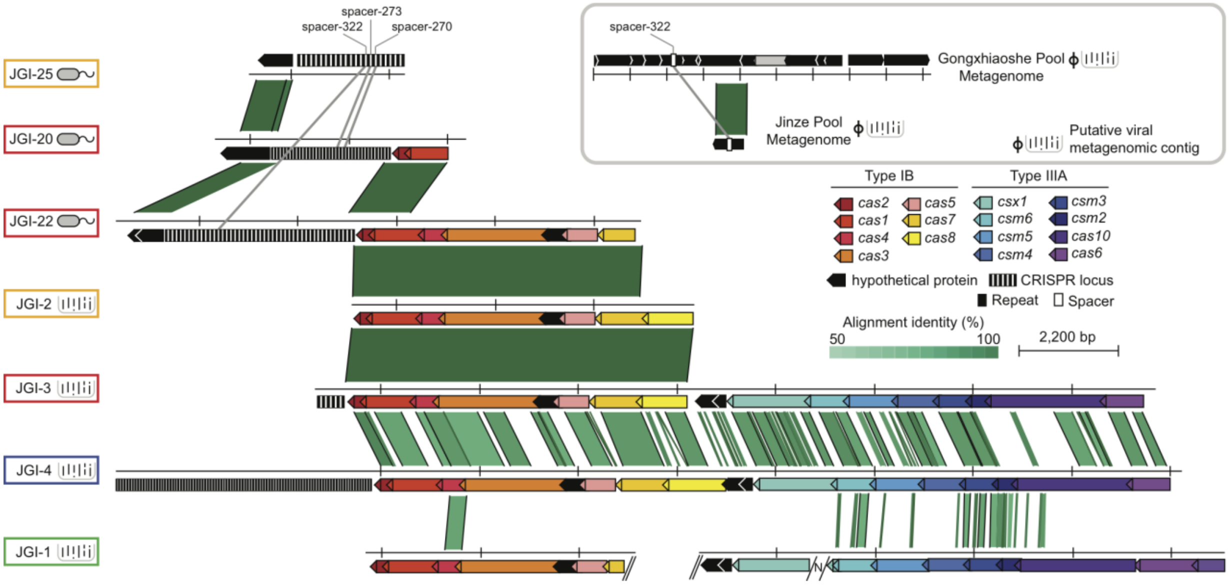

Supplementary Figure 5. Novel Type I-B/III-A CRISPR-*cas* fusion in '*Ca.* Kryptonia.' Alignment of CRISPR-Cas loci across '*Ca.* Kryptonia' GFMs and a subset of SAGs (11 out of 13 from Dewar Creek) with conserved gene synteny. For '*Ca.* Thermokryptus mobilis' JGI-1, the Cas gene complement was fragmented into two scaffolds. Putative genes encoding hypothetical proteins are shown in black, while CRISPR loci are denoted with repeats (black) and spacers (white). Conserved spacers are labeled with corresponding matches across genomes, as well as spacers identified in two putative viral contigs recovered from the Gongxiaoshe Pool and Jinze Pool metagenomes. Additional details for CRISPR repeats and identified spacers can be found in Supplementary Data 2.

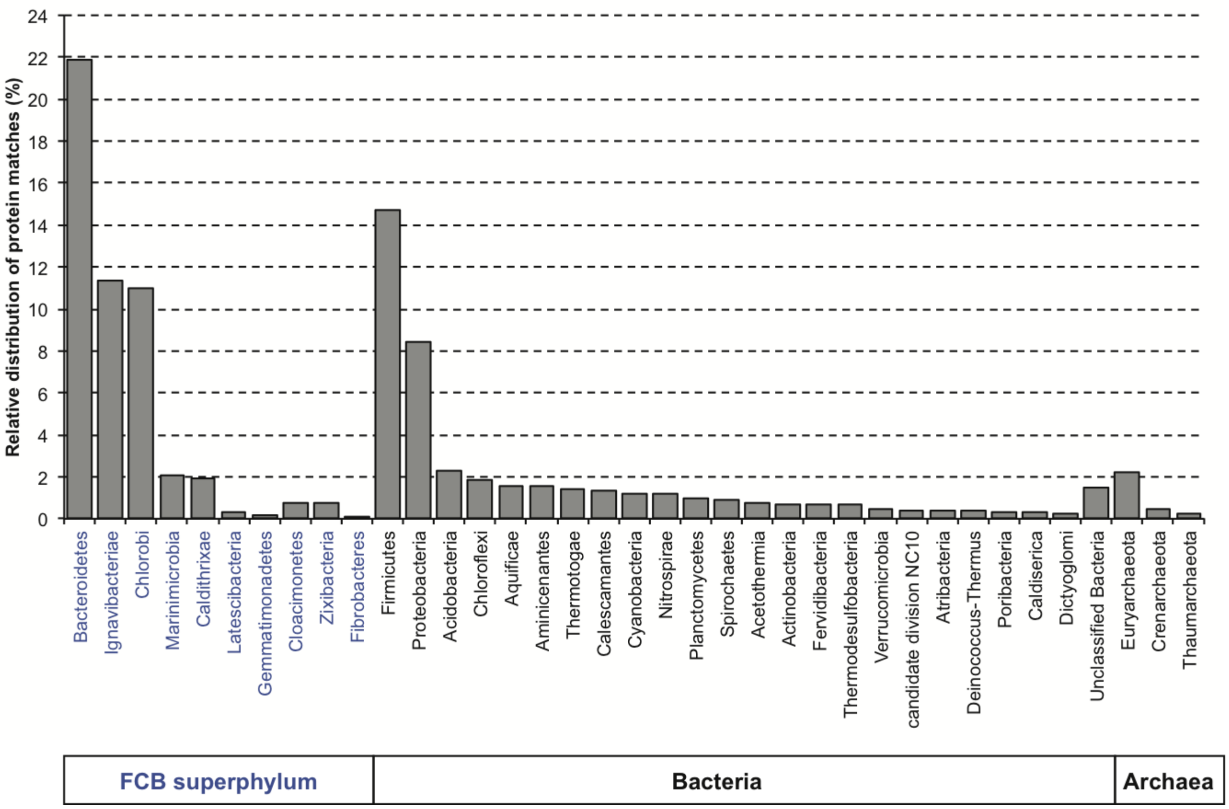

Supplementary Figure 6. Distribution of protein matches across all isolate genomes for all '*Ca.* Kryptonia' genomes. All predicted proteins from GFMs and SAGs were searched against a non-redundant database of high-quality isolate bacterial and archaeal reference genomes. A protein match was considered for proteins with ≥ 30% sequence identity across ≥ 50% of the query alignment length. Top matches were selected and phylogenetic affiliation at the phylum level was assigned.

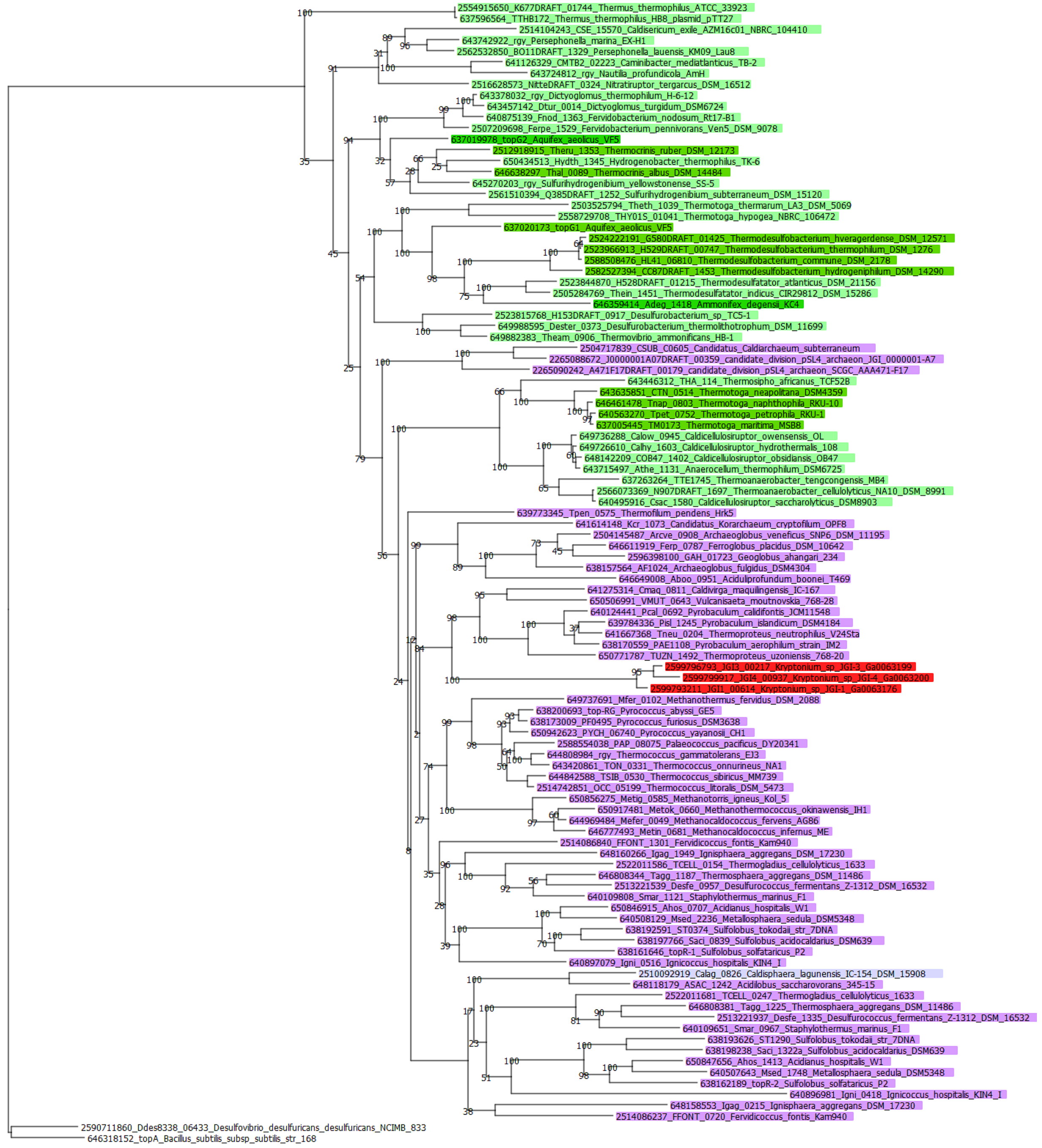

Supplementary Figure 7. Phylogenetic tree of reverse gyrase genes. Phylogenetic tree of reverse gyrase genes extracted from archaeal and bacterial genomes (selected as members of COG1110). Protein sequences were aligned using MAFFT and tree was constructed using RAxML[1]. The tree is rooted with topA sequences from *Bacillus subtilis* and *Desulfovibrio desulfuricans*. Sequences from '*Ca.* Kryptonia' genotypes are colored red, sequences from hyperthermophilic bacteria are colored dark green, sequences from extremely thermophilic bacteria are colored light green, sequences from hyperthermophilic archaea are colored dark amethyst, sequences from extremely thermophilic archaea are colored light amethyst.

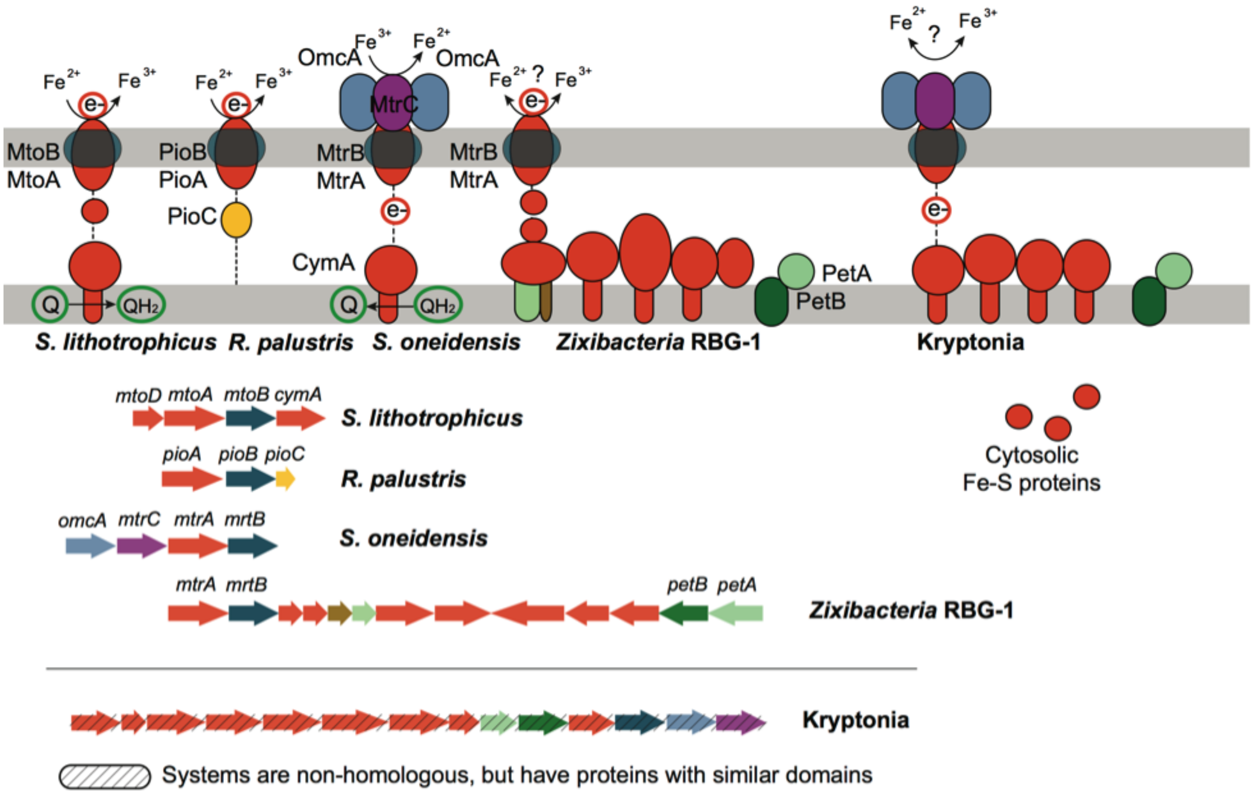

Supplementary Figure 8. Proposed iron respiration pathway in '*Ca.* Kryptonia.' Structural organization of proteins involved in iron oxidation from *Sideroxydans lithotrophicus* (Mto pathway), *Rhodopseudomonas palustris* (Pio pathway), iron reduction in *Shewanella oneidensis* (Mtr pathway), the putative iron respiration pathway in Zixibacteria RBG-1 (adapted from Castelle et al[2]), and the putative pathway in '*Ca.* Kryptonia.' Intracellular cytochromes are colored red, while extracellular cytochromes are colored blue (OmcA) and purple (MtrC). The gene cluster encoding the proteins putatively involved in the pathway is shown. Several multiheme periplasmic and inner membrane cytochrome *c* components are present, similar to *Candidatus* Zixibacteria RBG-1, yet conspicuously absent in the Zixibacteria and present in the '*Ca.* Kryptonia' genomes are extracellular cytochromes with canonical CXXCH motif predicted for haem-binding (pfam09698) and dodecaheme cytochrome *c* complex (pfam14522) (Supplementary Data 6).

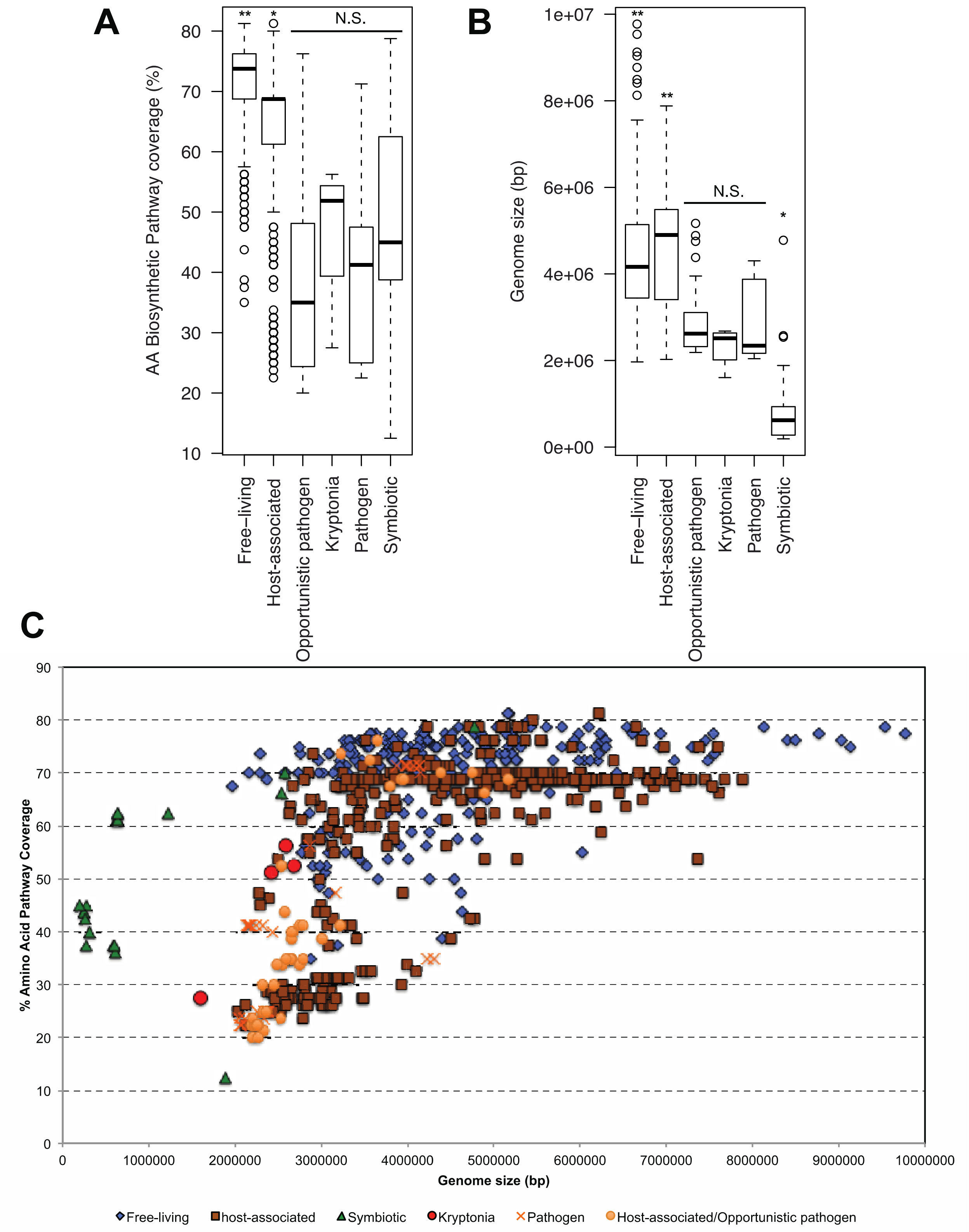

Supplementary Figure 9. Estimated coverage for amino acid biosynthetic pathways and genome size across members of the FCB superphylum. Predicted proteins required for all amino acid biosynthetic pathways were tallied, and represented

as a percentage of the total coverage for 759 FCB genomes. Biotic relationships were assigned from IMG metadata, and manually cross-validated and curated using literature citations. Verified relationship status was as follows: 176 free-living; 269 host-associated; 42 host-associated/opportunistic pathogen; 32 pathogen; and 20 symbiotic. (**A**) Amino acid biosynthetic pathway coverage. (**B**) Genome size distribution. (**C**) Scatterplot for all FCB genomes comparing pathway coverage and genome size. The nonparametric Wilcoxon rank sums test for multiple pairwise comparisons was employed to evaluate whether a difference in means was evident across biotic relationship categories. No significant differences were observed among '*Ca.* Kryptonia,' host-associated/opportunistic pathogens, pathogens, and symbiotic genomes for pathway coverage. Significant differences were observed between '*Ca.* Kryptonia' and free-living genomes ($p = 0.0023$), and '*Ca.* Kryptonia' and host-associated genomes ($p = 0.018$). Similarly for genome size, no significant differences were found among '*Ca.* Kryptonia,' host-associated/opportunistic pathogens, and pathogens, while significant differences were observed between '*Ca.* Kryptonia' and free-living genomes ($p = 0.00104$), '*Ca.* Kryptonia' and host-associated genomes ($p = 0.0015$), and '*Ca.* Kryptonia' and symbiotic genomes ($p = 0.013$). N.S. = not significant.

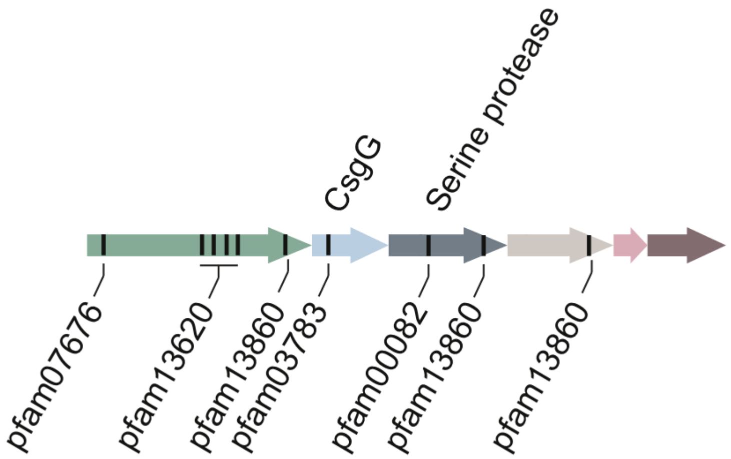

Supplementary Figure 10. Curli CsgG gene cluster for secretion of "functional amyloids." Representative six-gene cluster from *Armatimonadetes* bacterium GXS with the following annotations from left to right: WD40-like Beta Propeller Repeat/FlgD Ig-like domain/Carboxypeptidase regulatory-like domain protein (gene id: 2617203956); Curli biogenesis system outer membrane secretion channel CsgG (2617203957); Serine protease, subtilisin family (2617203958); FlgD Ig-like domain protein (2617203959); hypothetical protein (2617203960); and hypothetical protein (2617203961). Pfam ids are as follows: pfam13860, FlgD Ig-like domain; pfam00028, Subtilase family; pfam03783, Curli production assembly/transport component CsgG; pfam07676, WD40-like Beta Propeller Repeat; pfam13620, Carboxypeptidase regulatory-like domain. Homologous gene clusters were found in the *Armatimonadetes* bacterium DC, while the same region was fragmented in two contigs for *Armatimonadetes* bacterium GBS.

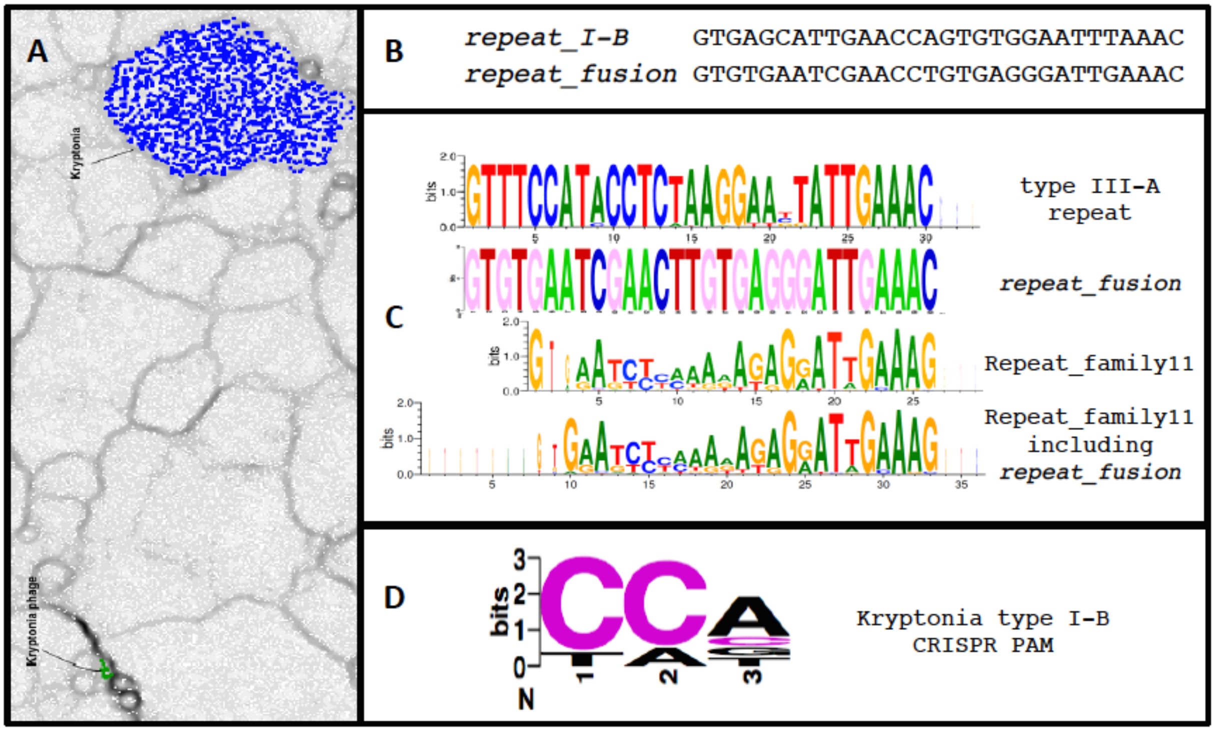

Supplementary Figure 11. Bacteriophage-related elements identified in '*Ca.* Kryptonia.' (A) Confined location of scaffolds assigned to '*Ca.* Kryptonia' phage (in green) using tetranucleotide-based binning methods (all '*Ca.* Kryptonia' ≥ 2 kb scaffolds are shown in blue). **(B)** '*Ca.* Kryptonia' CRISPR repeat sequences. **(C)** '*Ca.* Kryptonia' *repeat_fusion* sequence comparison with its closest repeat from the consensus sequences (type III-A repeat and family 11 repeat –according with data from CRISPR-map[3,4]). We can observe difference in the 5' end and strong sequence identity towards the 3' end. **(D)** Proto-spacer adjacent motif (PAM) found across all proto-spacers derived from '*Ca.* Kryptonia' type I-B CRISPR spacers.

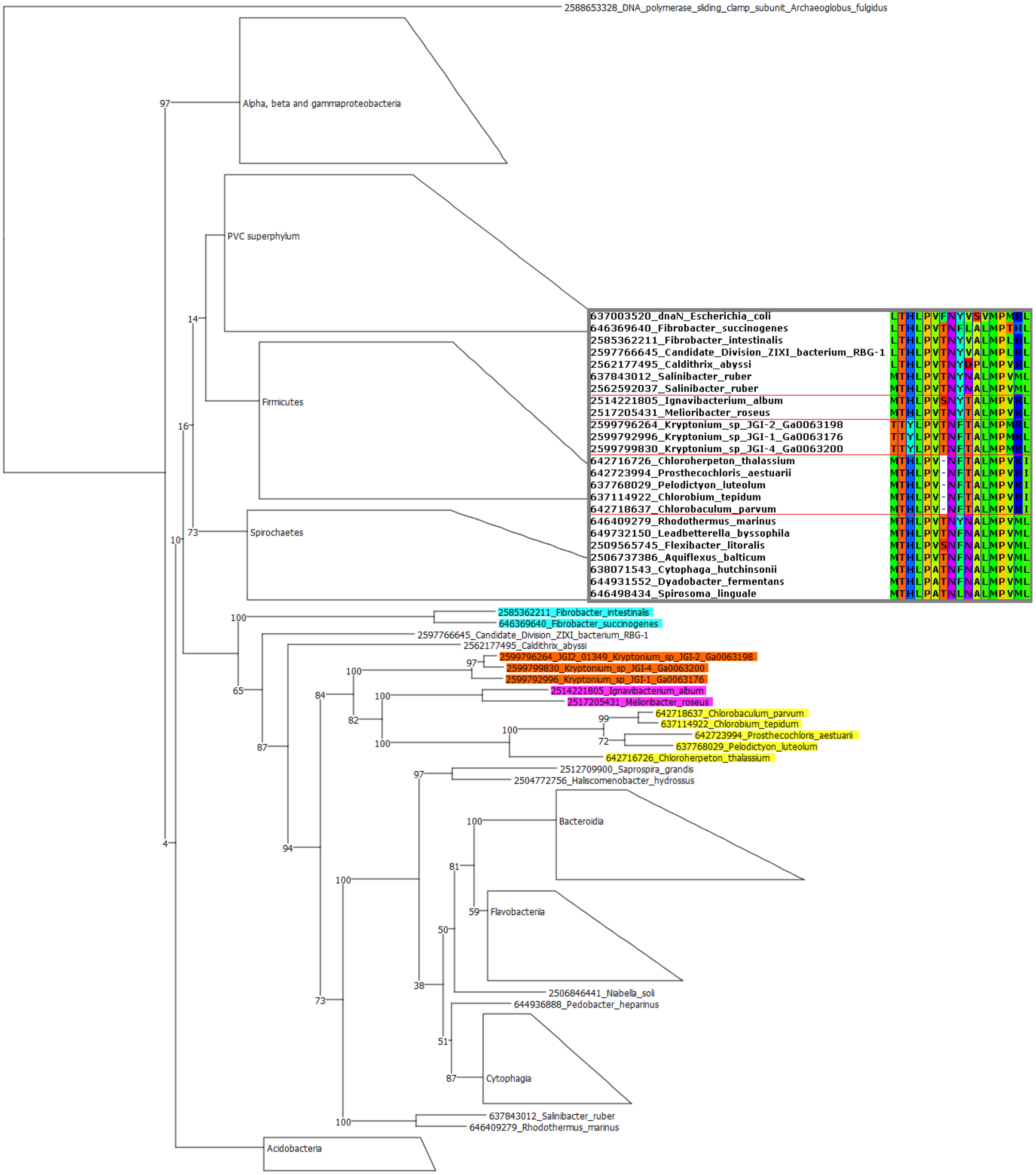

Supplementary Figure 12. Phylogram of dnaN orthologs. DNA polymerase III beta subunit or DNA polymerase III sliding clamp in Bacteria rooted by DNA polymerase sliding clamp sequence from *Archaeoglobus fulgidus*. Edges within the FCB superphylum are highlighted in bold. Sequences from *Fibrobacteres* are in turquoise, *Ignavibacteria* are in magenta, *Chlorobi* in yellow and ‘*Ca.* Kryptonia’ are in orange. Protein sequences were aligned using MAFFT, the tree was constructed using RAxML and visualized using Dendroscope. Inset shows an alignment of residues corresponding to L155, T172, H175, L177, P242, V247, F278, N320, Y323, V344, S346, V360, M362, P363, M364, R365, L366 of *E. coli* dnaN sequence, which were identified as a binding pocket for delta subunit and polIV DNA polymerase[5].

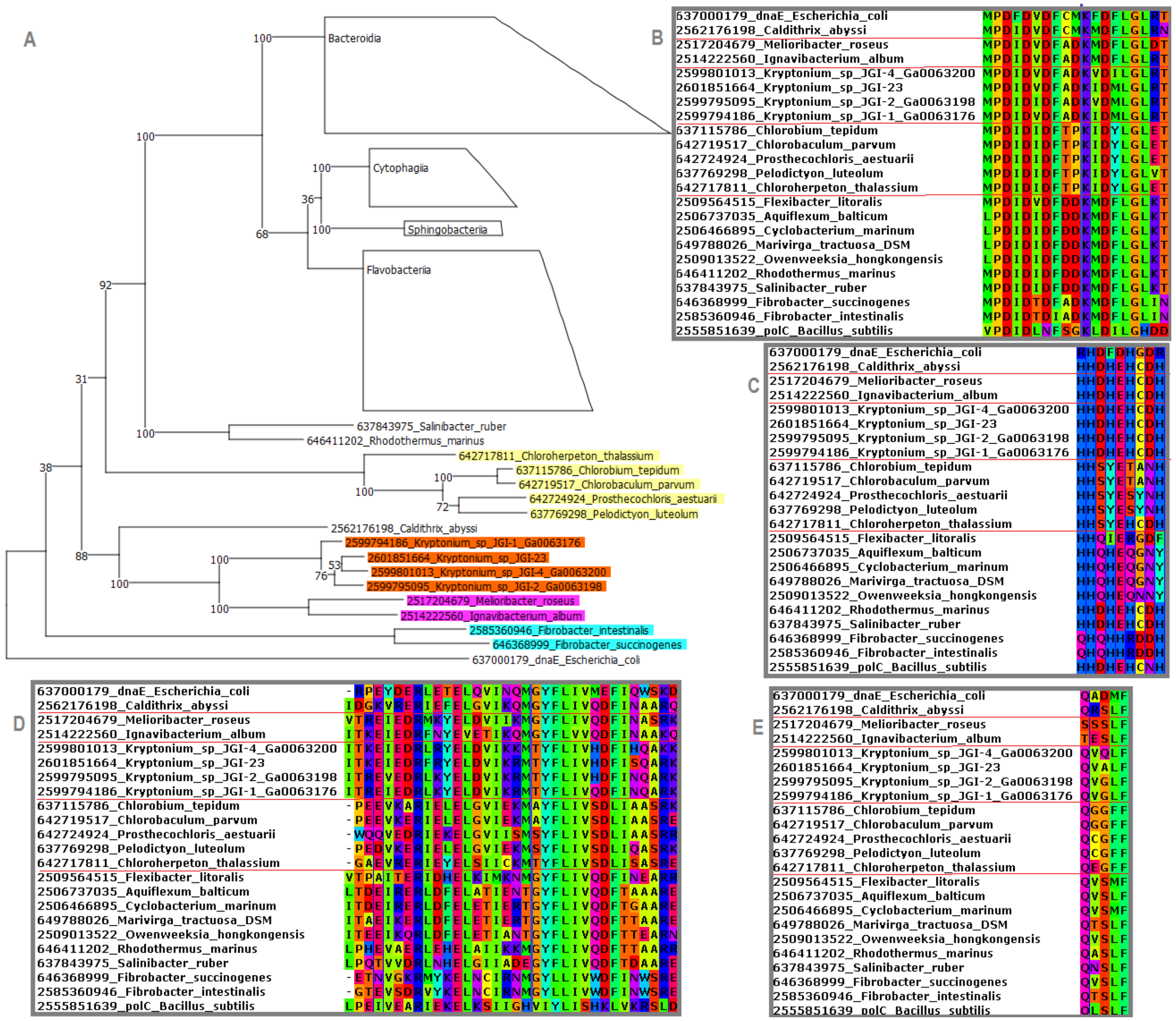

Supplementary Figure 13. Phylogenetic tree of dnaE1 orthologs. DNA polymerase III alpha subunit in members of the FCB superphylum rooted by dnaE from *E. coli* and alignments of active and binding sites. (**A**) Phylogram of dnaE1 proteins; sequences from *Fibrobacteres* are in turquoise, *Ignavibacteria* are in magenta, *Chlorobi* in yellow and '*Ca.* Kryptonia' are in orange. (**B**) Alignment of active site residues corresponding to residues 399-408 and 553-561 in *E. coli* dnaE[6]. (**C**) Alignment of an active site of PHP domain corresponding to residues R10, H12, D19, F44, D69, H83, G134, D201 and R203 in *E. coli* dnaE (*96*). (**D**) Alignment of epsilon-binding site corresponding to residues 311-335 and 339-353 in *E. coli* dnaE[7]. **E**. Alignment of clamp-binding site corresponding to residues 920-924 in *E. coli* dnaE[6].

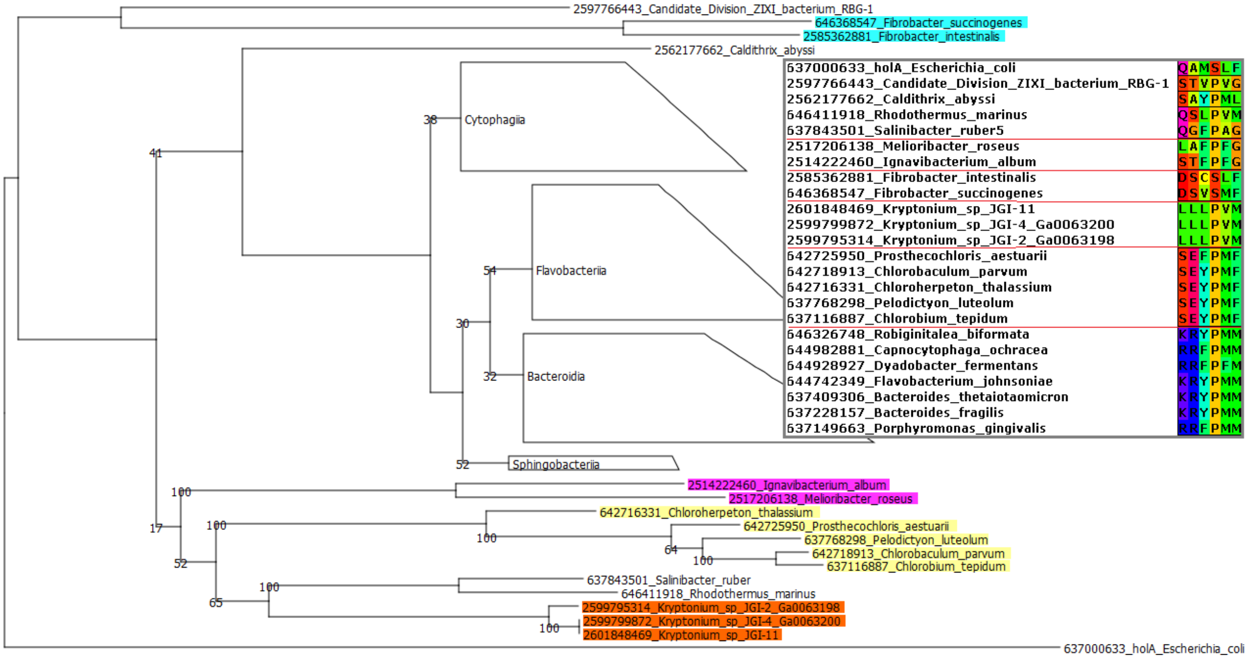

Supplementary Figure 14. Phylogenetic tree of holA orthologs. DNA polymerase III delta subunit in members of the FCB superphylum rooted by holA from *E. coli* and alignments of active and binding sites. In the phylogram of holA proteins sequences from *Fibrobacteres* are in turquoise, *Ignavibacteria* are in magenta, *Chlorobi* in yellow and '*Ca.* Kryptonia' are in orange. Inset: alignment of clamp-binding residues corresponding to 69-74 in *E. coli* holA[8].

Supplementary Table 1. ‘*Ca.* Kryptonia’ matches to previous SSU rRNA surveys. Datasets were collected as part of separate studies and searched against all ‘*Ca.* Kryptonia’ SSU rRNA sequences for similarity across ≥ 200 bp, and ≥ 95% ID. Publications and NCBI accession numbers are listed.

Location	Forward primer	Reverse primer	# Kryptonia matches	Total # Reads	% Kryptonia	NCBI SRA Accession	Citation
Great Boiling Springs, NV, USA	926F454TitFNew (AAACTYAAA**KG**AATTGRCGG)	1392R (ACGGGCGGTGTGT**R**C)	1	17,266	0.01	SRR611617	Peacock et al., 2013[9]
Gongxiaoshe Pool, China	515F (GTG**Y**CAGCMGCCGCGGTAA)	1391R (GACGGGCGGTGWGT**R**CA)	4,822	64,934	7.43	SRR611058, SRR611053 SRR611054, SRR611057, SRR611056, SRR611055, SRR611062, SRR611059, SRR611060, SRR611061	Hou et al., 2013[10]
Jinze Pool, China	515F (GTG**Y**CAGCMGCCGCGGTAA)	1391R (GACGGGCGGTGWGT**R**CA)	4	5,760	0.07	SRR611063, SRR611064	Hou et al., 2013[10]
Dewar Creek, British Columbia, Canada	926F (AAACTYAAA**KG**AATTGRCGG)	1392R (ACGGGCGGTGTGT**R**C)	13	7,321	0.18	SRP028305	Sharp et al., 2014[11]

Supplementary Table 2. Designation of new bacterial candidate phyla based on proposed thresholds by Yarza et al[12]. Distance matrices were calculated comparing all full-length sequences from '*Ca.* Kryptonia' against all full-length reference sequences classified by SILVA (NR_119) into the respective closest neighboring phyla. In each case, the median and minimum SSU rRNA sequence identities fell outside of the limits proposed by Yarza et al.[12] to define phylum-level clusters (highlighted in red bold). '*Ca.* Kryptonia' is therefore proposed as a new candidate phylum.

	**Number of references sequences**	**SSU sequence identity Median**	**SSU sequence identity Minimum**
**Minimum phylum boundaries proposed by Yarza et al.[12]**		**0.816**	**0.750**
Ca. Kryptonia vs Chlorobi (phylum)	838	**0.789**	**0.731**
Ca. Kryptonia vs Chlorobia (class)	450	**0.782**	**0.731**
Ca. Kryptonia vs Ignavibacteria (class)	388	**0.796**	**0.748**

Supplementary Table 3. Identification and assembly of genomes across four geothermal springs metagenomes and single cell genomes. (**A**) Assembly information for genomes extracted from metagenomes. (**B**) Assembly information for '*Ca.* Kryptonia' SAGs. Estimated genome completeness was based on marker gene set recovery. Tetranucleotide-based binning and signature-specific genome assembly procedures are described further in the Methods.

A. Assembly information for genomes extracted from metagenomes.

IMG Genome ID	Genome name	# Assembled Scaffolds	Genome Size (Mbp)	# Genes	Estimated completeness (%)	IMG Metagenome ID	Depth of Coverage from Metagenome
2599185253	*Ca.* Thermokryptus mobilis JGI-1 (GBS)	35	2.42	2319	96.18	3300000083	43.55
2599185254	*Ca.* Kryptobacter tengchongensis sp. JGI-2 (Jinze)	175	1.60	1659	67.99	3300000866	1.59
2599185255	*Ca.* Kryptobacter tengchongensis JGI-3 (Gongxiaoshe)	16	2.68	2405	95.65	3300000865	11.33
2599185256	*Ca.* Kryptonium thompsoni JGI-4 (Dewar Creek)	18	2.59	2353	95.63	3300002851	68.32
2616645014	*Armatimonadetes* bacterium DC	26	2.85	2630	93.06	3300002851	58.87
2616645015	*Armatimonadetes* bacterium GXS	46	2.92	2689	92.57	3300000865	11.20
2615840598	*Armatimonadetes* bacterium GBS	120	2.68	2545	90.74	3300000083	5.52

B. Assembly information for '*Ca.* Kryptonia' SAGs.

IMG Genome ID	Genome name	# Assembled Scaffolds	Genome Size (Mbp)	# Genes	Estimated completeness (%)
2600254939	*Ca.* Kryptonium thompsoni JGI-5 (Dewar Creek)	86	2.20	2,085	80.91
2600254940	*Ca.* Kryptonium thompsoni JGI-6 (Dewar Creek)	114	2.12	2,054	76.12
2600255340	*Ca.* Kryptonium thompsoni JGI-7 (Dewar Creek)	87	2.45	2,329	87.70
2600255341	*Ca.* Kryptonium thompsoni JGI-8 (Dewar Creek)	91	2.35	2,211	89.47
2600255342	*Ca.* Kryptonium thompsoni JGI-9 (Dewar Creek)	180	2.00	2,053	70.16
2600255343	*Ca.* Kryptonium thompsoni JGI-10 (Dewar Creek)	138	1.57	1,560	41.38
2600255344	*Ca.* Kryptonium thompsoni JGI-11 (Dewar Creek)	133	2.23	2,159	82.86
2600255348	*Ca.* Kryptonium thompsoni JGI-12 (Dewar Creek)	142	2.08	2,107	69.55
2600255349	*Ca.* Kryptonium thompsoni JGI-13 (Dewar Creek)	159	2.24	2,219	78.93
2600255396	*Ca.* Kryptonium thompsoni JGI-14 (Dewar Creek)	147	2.15	2,144	81.18
2600255388	*Ca.* Kryptonium thompsoni JGI-15 (Dewar Creek)	152	1.97	1,940	79.08
2600255391	*Ca.* Kryptonium thompsoni JGI-16 (Dewar Creek)	220	1.53	1,644	54.00
2600255395	*Ca.* Kryptonium thompsoni JGI-17 (Dewar Creek)	197	0.84	977	35.11
2600254941	*Ca.* Kryptobacter tengchongensis JGI-20 (Gongxiaoshe)	109	1.70	1,663	69.38
2600254942	*Ca.* Kryptobacter tengchongensis JGI-22 (Gongxiaoshe)	64	0.53	562	12.50
2600255345	*Ca.* Chrysopegis kryptomonas JGI-23 (Jinze)	43	2.07	1,980	72.41
2600255346	*Ca.* Kryptobacter tengchongensis JGI-24 (Jinze)	162	1.80	1,839	67.73
2600255347	*Ca.* Kryptobacter tengchongensis JGI-25 (Jinze)	198	1.67	1,676	61.77

Supplementary Table 4. Proposed names for candidate phylum '*Ca.* Kryptonia' genera and species.

Proposed name and etymology of Candidate phylum	Proposed Candidatus species	Type species location	#SAGs/ GFMs
**"Kryptonia"** Kryp.to'ni.a. A higher taxonomic unit comprising the genera Kryptonium, Kryptobacter, Thermokryptus, and Chrysopegis -krypton Gr. adj., hidden	***Candidatus* Kryptonium thompsoni** Kryp.to'ni.um. Gr. adj. krypton, hidden; Gr. n. bios, life; N.L. neut. n. Kryptonium a hidden life form. thomp.son'i N. L. adj. after David Thompson, explorer of the region around Dewar Creek	**Dewar Creek**	**13/1**
	***Candidatus* Kryptobacter tengchongensis** Kryp.to.bac'ter. Gr. adj. kryptos, hidden; N.L. n. bacter, rod; N.L. masc. n. Kryptobacter a hidden rod teng.chong.en'sis. N. L. adj. from Tengchong County, China.	**Jinze, Gongxiaoshe**	**4/2**
	***Candidatus* Thermokryptus mobilis** Thermo.kryp'tus. Gr. adj. thermos, hot; Gr. adj. kryptos, hidden; N.L. masc. n. Thermokryptus a (bacterium from a) hidden hot place mo'bi.lis. L. adj. mobilis, motile, moving	**Great Boiling Spring**	**0/1**
	***Candidatus* Chrysopegis kryptomonas** Chryso.pe'gis. Gr. adj. chrysos, golden; Gr. n. pege, a spring; N.L. gen. n. Chrysopegis, from a golden spring or pool kryp.to.mo'nas. Gr. adj. kryptos, hidden; Gr. fem. n. monas, a single unit; N.L. fem. n. kryptomonas a hidden single cell	**Jinze**	**1/0**

Supplementary Table 5. Hot spring metagenomes included in the SSU rRNA-targeted assembly.

IMG Genome ID	Genome Name / Sample Name	Short name	Sequencing Center	Assembled size, Mb
2053563014	Hot spring sediment microbial communities from Great Boiling Spring, Nevada - surface sediment (Surface sediment)	GBS surface sediment	DOE Joint Genome Institute (JGI)	48.2
2100351008	Hot spring microbial communities from Beowulf Spring, Yellowstone National Park, Wyoming, USA - YNP_Beowulf Spring_E (YNP_Beowulf Spring_E)	Beowulf Spring E	DOE Joint Genome Institute (JGI)	66.7
2119805007	Hot spring microbial communities from Beowulf Spring, Yellowstone National Park, Wyoming, USA - YNP_Beowulf Spring_D (YNP_Beowulf Spring_D)	Beowulf Spring D	DOE Joint Genome Institute (JGI)	60.7
3300000082	Hot spring sediment microbial communities from Great Boiling Spring, Nevada - Cellulolytic enrichment Sediment 77C (GBS Cellulolytic enrichment 77S sediment, Feb 2012 assem)	S77sediment	DOE Joint Genome Institute (JGI)	79
3300000083	Hot spring microbial communities from Great Boiling Spring, Nevada - cellulolytic enrichment S 77C (GBS Cellulolytic enrichment S 77C water, Feb 2012 assem)	S77water	DOE Joint Genome Institute (JGI)	63.3
3300000084	Hot spring sediment microbial communities from Great Boiling Spring, Nevada - Cellulolytic enrichment CS 85C (GBS Cellulolytic enrichment CS 85C sediment, Feb 2012 assem)	CS85sediment	DOE Joint Genome Institute (JGI)	56.1
3300000106	Hot spring sediment microbial communities from Great Boiling Spring, Nevada - cellulolytic enrichment CS 77C (GBS Cellulolytic enrichment CS 77C sediment, Combined June 2011 assem)	CS77sediment	DOE Joint Genome Institute (JGI)	83.7
3300000340	Hot spring microbial communities from Echinus Geyser, Yellowstone National Park, USA - transect B T=78-80 C (Echinus Geyser transect B T=78-80 C, June 2012 Assem)	Echinus 78-80	DOE Joint Genome Institute (JGI)	54.4
3300000341	Hot spring microbial communities from One Hundred Spring Plain, Yellowstone National Park, USA (One Hundred Spring Plain T=66-70, June 2012 Assem)	One Hundred Spring Plain 66-70	DOE Joint Genome Institute (JGI)	50.1
3300000342	Hot spring microbial communities from One Hundred Spring Plain, Yellowstone National Park, USA - T=74-76 (One Hundred Spring Plain T=74-76, June 2012 Assem)	One Hundred Spring Plain 74-76	DOE Joint Genome Institute (JGI)	53.8
3300000345	Hot spring microbial communities from Octopus Spring Streamers, Yellowstone National Park, USA T=80-84 (Octopus Spring Streamers T=80-84, June 2012 Assem)	Octopus Streamers	DOE Joint Genome Institute (JGI)	49.1
3300001337	Hypersaline microbial mat communities from Conch Spring, Yellowstone National Park, USA - CON_C (Conch CON_C Metagenome, ASSEMBLY_DATE=20130420)	Conch Spring	DOE Joint Genome Institute (JGI)	62.8

IMG Genome ID	Genome Name / Sample Name	Short name	Sequencing Center	Assembled size, Mb
3300001340	Hypersaline microbial mat communities from Yellowstone National Park, USA - RS3B (RS3B Metagenome, ASSEMBLY_DATE=20130423)	RS3B	DOE Joint Genome Institute (JGI)	264.1
3300001446	Hypersaline microbial mat communities from Washburn Spring, Yellowstone National Park, USA - WS (Washburn Spring WS Metagenome, ASSEMBLY_DATE=20130521)	Washburn Spring	DOE Joint Genome Institute (JGI)	287.4
3300003598	Hypersaline microbial mat communities from Yellowstone National Park, Wyoming, USA - Perpetual Spouter B (PS_B) MetaG (Hypersaline microbial mat communities from Yellowstone National Park, Wyoming, USA - Perpetual Spouter B (PS_B) MetaG, ASSEMBLY_DATE=20141005)	Perpetual Spouter B	DOE Joint Genome Institute (JGI)	73.2
3300003603	Hypersaline microbial mat communities from Yellowstone National Park, Wyoming, USA - Perpetual Spouter C (PS_C) MetaG (Hypersaline microbial mat communities from Yellowstone National Park, Wyoming, USA - Perpetual Spouter C (PS_C) MetaG, ASSEMBLY_DATE=20141004)	Perpetual Spouter C	DOE Joint Genome Institute (JGI)	79.8
3300003604	Hypersaline microbial mat communities from Yellowstone National Park, Wyoming, USA - Perpetual Spouter A (PS_A) MetaG (Hypersaline microbial mat communities from Yellowstone National Park, Wyoming, USA - Perpetual Spouter A (PS_A) MetaG, ASSEMBLY_DATE=20141005)	Perpetual Spouter A	DOE Joint Genome Institute (JGI)	62.6
3300003605	Hypersaline microbial mat communities from Yellowstone National Park, Wyoming, USA - Fairy Falls C (FF_Mn_C) (Hypersaline microbial mat communities from Yellowstone National Park, Wyoming, USA - Fairy Falls C (FF_Mn_C) MetaG, ASSEMBLY_DATE=20141008)	Fairy Falls C	DOE Joint Genome Institute (JGI)	114.6
3300003606	Hypersaline microbial mat communities from Yellowstone National Park, Wyoming, USA - Fairy Falls B (FF_Mn_B) (Hypersaline microbial mat communities from Yellowstone National Park, Wyoming, USA - Fairy Falls B (FF_Mn_B) MetaG, ASSEMBLY_DATE=20141007)	Fairy Falls B	DOE Joint Genome Institute (JGI)	111.4

Supplementary Table 6. SSU rRNA clusters at 90% identity most correlated with '*Ca.* Kryptonia' genotypes.

Cluster ID	Lineage	SILVA accession numbers	Spearman's rank-order correlation
3107	Bacteria; Armatimonadetes	GU437340, DQ645244, HM640993, DQ645248	0.82
4987	Bacteria; Chloroflexi; Anaerolineae; Anaerolineales; Anaerolineaceae	GU437647, FN545868	0.81
3631	Bacteria; Deinococcus-Thermus; Deinococci; Thermales; Thermaceae; Thermus	AY082363, AIJQ01000002, AF407694, FR749952, KC852874, CP001962, GU437245, ARDI01000014, AB745045	0.75
3283	Bacteria; GAL08	AP011670	0.75
3736	Bacteria; Chloroflexi; Thermomicrobia; Thermomicrobiales; Thermomicrobiaceae	M34115, FN545867, HE794997, DQ324896	0.75
5702	Bacteria; Thermotogae; Thermotogae; Thermotogales; Thermotogaceae; EM3	AF407680, AF018187, HM448393	0.73
3691	Bacteria; Chloroflexi; S085	FN545887	0.72
4418	Bacteria; S2R-29	HM448388	0.71
8304	Archaea; Thaumarchaeota; Thaumarchaeota Incertae Sedis; Unknown Order; Unknown Family; Candidatus Nitrosocaldus	AP011903, JF262244, DQ490011, EU239993	0.7
3610	Bacteria; Chlorobi; Chlorobia; Chlorobiales; OPB56	AP011715	0.68
4986	Bacteria; Candidate division OD1	EU156156	0.67
3187	Bacteria; Firmicutes; Clostridia; Thermoanaerobacterales; Family XVII; Candidatus Fervidibacter	AWNZ01000028	0.66
5351	Bacteria; Candidate division OD1	AF352532, EU924256	0.62
3522	Bacteria; Aquificae; Aquificae; Aquificales; Aquificaceae	CP007028, AY082371, CP001931, AF018191, AM937264, AB183857, HM448227, FM164962, FM164952, JF501079, DQ924674, DQ833792, AY882730, DQ179021	0.6
8838	Archaea; Thaumarchaeota; Terrestrial Hot Spring Gp (THSCG); Unknown Order; Unknown Family; Candidatus Caldiarchaeum	AP011786, JN881568, EU635915, AB213053, HM448147, DQ243752	0.57
4353	Bacteria; Acetothermia (Candidate division KB1)	AP011801, EU635953, HM448253, FJ821658	0.54
4395	Bacteria; Thermotogae; Thermotogae; Thermotogales; Thermotogaceae; EM3	HM448268	0.53
29809	Bacteria	None	0.52
4318	Bacteria; Firmicutes; Clostridia; Thermoanaerobacterales	ASMU01000034	0.51

Supplementary Table 7. Predicted proteins in '*Ca.* Kryptonia' and *Thermus* spp. for enzymatic components of the denitrification pathway. Both *Thermus* genomes were from cultivated microbes isolated from the Great Boiling Spring[13]. IMG genome ids are in parentheses.

Function ID	Name	Thermokryptus mobilis JGI-1 (2599185253)	*Thermus oshimai* JL-2 (250850104)	*Thermus thermophilus* JL-18 (2508501108)
KO:K00370	nitrate reductase alpha subunit [EC:1.7.99.4] (narG)	0	1	1
KO:K00371	nitrate reductase beta subunit [EC:1.7.99.4] (narH)	0	1	1
KO:K00373	nitrate reductase delta subunit (narJ)	0	1	1
KO:K00374	nitrate reductase gamma subunit [EC:1.7.99.4] (narI)	0	1	1
KO:K04561	nitric oxide reductase subunit B [EC:1.7.2.5] (norB)	0	1	1
KO:K02305	nitric oxide reductase subunit C (norC)	0	1	1
KO:K15864	nitrite reductase (NO-forming) / hydroxylamine reductase [EC:1.7.2.1 1.7.99.1] (nirS)	0	1	1
KO:K00376	nitrous-oxide reductase [EC:1.7.2.4] (nosZ)	1	0	0
KO:K02567	periplasmic nitrate reductase NapA [EC:1.7.99.4] (napA)	1	0	0

Supplementary Notes

Supplementary Note 1. CRISPR-Cas

We used 99 CRISPR-associated (*cas*) gene sequence alignments and hidden Markov models from the TIGRFAM database (originally built by Haft *et al*.[14] and later expanded by Zhang *et al*.[15]) to precisely find and identify Cas family members within the scaffolds of the '*Ca.* Kryptonia' genomes. We recovered and classified the corresponding CRISPR type for complete and partial CRISPR-Cas loci in all genomes following the unified CRISPR classification from 2011 (ref.[16]).

In the Dewar Creek genomes (GFM JGI-4, and SAGs JGI-5 – JGI-17), we identified the presence of three CRISPR repeat-spacer arrays located at different genomic loci, two of them containing associated *cas* genes (Supplementary Data 2). We classified one of the CRISPR-Cas systems as type I-B that contained 34 spacers, and harbored a conserved repeat (here referred as *repeat_I-B*; Supplementary Fig. 11) that was also found in the separate array (31 spacers) without *cas* genes associated. The second *cas* locus was found to be a highly unusual completed fusion between two main CRISPR-Cas types (type I and III; subtypes I-B and III-A). This fusion shares the genes responsible for spacer acquisition (*cas*2 and *cas*1) and crRNA processing (*cas*6), containing also the whole set of essential genes needed in the CRISPR ribonucleoprotein (crRNP) complex (*cas*5, *cas*7, and *cas*8 for the type I-B; and *csm2*, *csm*3, *csm*4, *csm*5, and *cas*10 for the type III-A) and the target degradation (*cas*3, and *csm*6 for type I-B and type III-A, respectively)[17,18]. Additionally, the fusion integrates a *cas*4 gene (recently related with possible programmed cell death in bacteria[19]), a *csx*1 gene (usually related in subtype III-U), and three hypothetical genes without any known domain assigned (Supplementary Fig. 5). Interestingly, the CRISPR-Cas fusion array was associated with a singular hybrid repeat (from now on *repeat_fusion*) where the 5' end of the sequence agrees better with type I repeat patterns and the 3' end matches with type III-B repeat pattern (Supplementary Fig. 11), and as many as 150 spacer sequences (Supplementary Fig. 5). After the reconstruction of the three repeat-spacer arrays using GFM JGI-4 and the corresponding SAGs, we did not observe any spacer multi-variation across the Dewar Creek genomes, indicating that we might have collected a clonal population or maybe these CRISPR-Cas system are not dynamic in terms of spacer acquisition (Supplementary Data 2).

We used the combination of the 3 genomes found in the Gongxiaoshe sample (GFM JGI-3, SAG JGI-20, and SAG JGI-22) to identify the same type I-B/III-A fusion (Supplementary Fig. 5). This hybrid *cas* gene arrangement was associated with the same *repeat_fusion* sequence found in Dewar Creek genomes and we recovered a distinct spacer set across the genomes. The same occurred in the *repeat_fusion*-spacer locus without any *cas* genes found in GFM-JGI-3 and SAG JGI-20 (Supplementary Fig. 5). This finding clearly indicated activity in the spacer acquisition for this CRISPR system. Additionally, we detected repeat-spacer arrays containing *repeat_I-B* in all Gongxiaoshe genomes. These arrays also presented multi-variation in the spacer content although they were either not associated with *cas* genes or associated with an incomplete array of *cas* genes (assigned to type-I due to the presence of the marker gene *cas*3).

We used the above mentioned hybrid *cas* gene arrangement as an anchor to reconstruct the fragmented CRISPR-Cas system of Jinze genomes GFM JGI-2 (Supplementary Fig. 5) and SAG JGI-25. We were unable to recruit the corresponding *cas* genes associated with the hybrid system in SAGs JGI-23 and JGI-24. We detected a total number of 2, 126, 71, and 53 spacers connected with the *repeat_fusion* across 1, 4, 2 and 4 different loci for the GFM JGI-2, SAG JGI-23, SAG JGI-24, and SAG JGI-25, respectively. All the spacers detected were unique for each genome, strongly suggesting a dynamic and active CRISPR-Cas system. Moreover, the four Jinze genomes presented an almost identical *repeat_I-B* (one C:A SNP in position 6 of the repeat) to the repeat in the genomes from Dewar Creek and Gongxiaoshe. We were able to recruit the corresponding type I-B *cas* genes in the 3 Jinze SAGs but not in the GFM JGI-2 genome. In total, we collected unique sets of 2, 18, 9 and 57 spacers associated with *repeat_I-B* for Jinze genomes GFM JGI-2, SAG JGI-23, SAG JGI-24, and SAG JGI-25, respectively, also hinting a dynamic activity of this CRISPR-Cas system.

From the GBS sample, where we have a single genome (GFM JGI-1), we only identified those *cas* genes present in the type I-B/III-A fusion in two separate scaffolds (Supplementary Fig. 5). Due to the fragmentation of GFM JGI-1 genome we were unable to recruit any spacers associated using the assembled data. We used then a different algorithm (CRISPR assembler "Crass"[20]) that uses directly the raw reads to search for CRISPR spacers and repeats (see Methods). We recovered in total 56 spacers associated with the GFM JGI-1 sample.

We used the IMG/M database to search for high sequence identity of the total collection of 795 unique spacer groups that we clustered from all '*Ca.* Kryptonia' genomes. After removing self-hits (spacers from its own source), we exclusively detected 29 perfect spacer hits (for both sequence identity and length) across five distinct scenarios: (i) 3 spacers from Jinze '*Ca.* Kryptonia' genomes hit Jinze metagenomic scaffolds, (ii) 5 spacers from Gongxiaoshe '*Ca.* Kryptonia' genomes hit Gongxiaoshe metagenomic scaffolds, (iii) 15 spacers from Jinze '*Ca.* Kryptonia' genomes hit Gongxiaoshe metagenomic scaffolds, (iv) 4 spacers from Gongxiaoshe '*Ca.* Kryptonia' genomes hit Jinze metagenomic scaffolds, and (v) 2 spacers from GBS JGI-1 genome hit GBS virome sample (Supplementary Data 3). All of the targeted scaffolds (ranging from 0.2-7.2 Kbp) were assigned to phage or "unknown" based on their gene content (Supplementary Data 5). We hypothesize that most of these scaffolds are viral also based on the number of hypothetical genes, and their length and directionality[21]. We found 11 shared spacers between Jinze and Gongxiaoshe genomes (Supplementary Data 2). Interestingly, one of these spacers hit the same phage scaffold detected in both metagenomes (Supplementary Fig. 5) indicating the presence of the same infective phage in these ecosystems. We also considered 41 other non-perfect spacer matches (at least 90% spacer identity over 91% sequence length) within the same above-mentioned scenarios (Supplementary Data 3).

Notably, we observed no perfect hits (for both sequence and length) for any of the '*Ca.* Kryptonia' spacer groups recruited against any other metagenome. Across all samples used in this comparison we detected a 92% identity (33/36 nt) over 100% length match of a spacer (group_302) from Gongxiaoshe SAG JGI-22 genome with 2 similar scaffolds detected in Yellowstone National Park samples[22]. This may indicate the

presence of similar players across similar environments, although the scaffold gene content did not provide sufficient phylogenetic information for taxonomic assignment. Moreover, we noticed a striking spacer match of 89% identity (32/36 nt) over 100% of the sequence length from a Gongxiaoshe spacer (group_291) to a scaffold from a virome study in the GBS location harboring a terminase gene. Lastly, we were able to connect spacers from Dewar Creek and Jinze to spacers coming from the GBS metagenome (other than JGI-1). Based on the detection of all these proto-spacers (original spacer sequence location: e.g phage), we identified a PAM (proto-spacer adjacent motif) for the type I-B CRISPR system that agreed with the requirement for this type (CCn-Protospacer; Supplementary Fig. 11). In the case of the type I-B/III-A CRISPR-Cas fusion, we were unable to detect any PAM, in accordance with the lack of PAM for all type III system described so far[16].

Taken together, these data indicate the activity of the CRISPR systems of '*Ca.* Kryptonia' and strongly support the presence of the same or very similar '*Ca.* Kryptonia' infecting phages across similar environments.

Supplementary Note 2. Metabolic and functional features of *Candidatus* Kryptonia

Extreme thermophilic adaptation

Reverse gyrase is an enzyme introducing positive supercoils into circular DNA, which has been strongly associated with extreme thermophilic and hyperthermophilic lifestyle, i. e. optimal growth temperature of above 70°C[23]. A reverse gyrase gene was found in representatives of all '*Ca.* Kryptonia' genotypes suggesting that most if not all members of this candidate lineage are extreme thermophiles or hyperthermophiles. Since no other lineages in the FCB superphylum harbor this gene, we hypothesized that it has been acquired by horizontal transfer from other extreme thermophilic or hyperthermophilic microbes. Alignment of reverse gyrase sequences and phylogenetic tree reconstruction placed '*Ca.* Kryptonia' sequences into a branch distinct from all other bacterial sequences and pointed to crenarchaeotes of the *Thermoproteales* order as the likely source of the reverse gyrase gene in '*Ca.* Kryptonia' (Supplementary Fig. 7). This suggests an event independent of proposed horizontal gene transfer of reverse gyrase from archaea into the ancestors of two bacterial phyla with hyperthermophilic representatives, *Aquificae* and *Thermotogae*[24]. We used the GC content of the '*Ca.* Kryptonia' 16S rRNA gene to estimate its optimal growth temperature[25]. All '*Ca.* Kryptonia' genotypes had 16S genes with GC content of 61%, which corresponds to the predicted optimal growth temperature between 66°C and 76°C. This range is in agreement with the temperatures of geothermal spring sites in which '*Ca.* Kryptonia' has been found.

Another potential signature of extreme thermophilic or hyperthermophilic lifestyle of '*Ca.* Kryptonia' is the absence of dihydrouridine synthase genes in all '*Ca.* Kryptonia' genotypes. These enzymes perform posttranscriptional reduction of uridines in RNAs and are common in bacteria, eukaryotes and euryarchaeota[26]. Since dihydrouridine ring is not aromatic and non-planar, uridine reduction prevents stacking interactions with other nucleosides, thereby increasing RNA flexibility. Psychrophilic bacteria and archaea are known to have many dihydrouridine modifications in their tRNAs[27], whereas thermophilic tRNAs often lack dihydrouridine and carry other types of modifications, such as methylated nucleosides, which stabilize their secondary structure[28]. A diverse

complement of potential tRNA methyltransferases was identified in different '*Ca.* Kryptonia' genomes including up to four copies of SpoU family methylases (identified by matches to Pfam family PF00588), which include bacterial tRNA methyltransferases trmH, trmJ and trmL[29-31]. Only two of '*Ca.* Kryptonia' SpoU-like methylases (exemplified by JGI1_01862 and JGI1_00473 from GFM-1) have putative orthologs in other members of the FCB superphylum suggesting that the remaining enzymes may be involved in thermophilic adaptation.

Extreme thermophilic and hyperthermophilic bacteria and archaea produce a variety of compatible solutes, some of which have not only osmoprotective, but also thermoprotective properties[32]. These include amino acids and derivatives, such as aspartate and beta-glutamate, sugars and derivatives, such as trehalose and mannosylglycerate, and phosphorylated compounds, such as diglycerol phosphate and di-*myo*-inositol phosphate. All '*Ca.* Kryptonia' genomes have myo-inositol phosphate synthase gene (exemplified by JGI1_02220 from GFM-1), but no di-myo-inositol phosphate synthase gene was found. However, the genomes from all locations encode multiple glycosyltransferase genes, most of them of unknown specificity (see below). Some of these glycosyltransferases could be responsible for synthesis of sugar-derived compatible solutes. In addition, genomes from the Dewar Creek hot spring harbor a bifunctional trehalose-phosphate synthase/trehalose-bisphosphatase (exemplified by JGI4_00881 in GFM-4), which produces trehalose, a compatible solute with some thermostabilizing properties[33].

Ca. *Kryptonia replication machinery.*

Whereas multiple lines of evidence supported affiliation of '*Ca.* Kryptonia' genotypes within the FCB superphylum, they did not stably associate with any established phylum within this group. Since members of the FCB superphylum are extremely diverse and adapted to a broad variety of lifestyles, ranging from obligate photoautotrophy[34] to obligate endosymbiosis[35], we analyzed replication machinery of '*Ca.* Kryptonia' and its FCB relatives in an attempt to refine its placement within the superphylum. Bacterial replication is an essential process carried out by protein apparatus well conserved over large phylogenetic distances[36]. However, since bacterial replication machinery is distinct from that of archaea and eukaryotes, replication proteins were not part of the universal single-copy gene set. Orthologs of *E. coli* dnaE, dnaN and holA coding for DNA polymerase III alpha, beta and delta subunits, respectively, were collected from '*Ca.* Kryptonia' and a set of reference FCB genomes in IMG. These were identified as proteins with hits to signature Pfam models (PF07733, PF02767 and PF13177) that were subjected to further refinement based on bi-directional best hits. Protein sequences were aligned, active site residues were analyzed and phylogenetic trees were constructed (Supplementary Figs. 12-14). '*Ca.* Kryptonia' sequences in dnaE, dnaN and holA trees were again robustly associated with the FCB superphylum, but their specific affiliation was different. In the dnaN tree (Supplementary Fig. 12), '*Ca.* Kryptonia' appeared as a basal lineage to the *Ignavibacteria* and *Chlorobi*; in the dnaE tree (Supplementary Fig. 13), '*Ca.* Kryptonia' are a sister lineage to *Ignavibacteria*; and in the holA tree (Supplementary Fig. 14), '*Ca.* Kryptonia' does not cluster with either phylum. Analysis of active site residues in DNA polymerase subunits also suggests that '*Ca.* Kryptonia' is a distinct lineage within the FCB superphylum. For instance, in the

dnaN binding pocket a histidine residue corresponding to H175 in *E. coli* is nearly universally conserved in FCB sequences, but it is replaced with tyrosine in '*Ca.* Kryptonia' (Supplementary Fig. 12). In addition, clamp-binding pockets in '*Ca.* Kryptonia' dnaE and holA are poorly conserved. The consensus sequence of clamp-binding site in '*Ca.* Kryptonia' dnaE is QV[QAG]LF, which is similar to the *E. coli* consensus for clamp-binding sites (QL[S/D]LF) (Supplementary Fig. 13D). However, the consensus sequence of clamp-binding site in holA is LLLPVM, which is different both from '*Ca.* Kryptonia' dnaE consensus and from consensus clamp-binding sites in other members of FCB superphylum (Supplementary Fig. 14). Other potential clamp-binding proteins in '*Ca.* Kryptonia' include polB (DNA polymerase II) and error-prone DNA polymerase detected in '*Ca.* Kryptobacter tengchongensis' JGI-24 and '*Ca.* Chrysopegis kryptomonas' JGI-23. Consensus sequence of the clamp binding site in the former is DG[LI]NF and QQHLF in the latter, which is reminiscent of '*Ca.* Kryptonia' dnaE clamp-binding site and *E. coli* consensus clamp-binding site. Although the lack of sequence conservation between different clamp-binding sites does not always indicate the difference in interaction mechanism[8], it is uncommon in the representatives of the FCB superphylum and supports '*Ca.* Kryptonia' distinct position within this group.

Proteases

A putative cysteine endopeptidase from the peptidase family C25 was recovered and found to be distantly related to the well-characterized gingipains from the anaerobic periodontal pathogen *Porphyromonas gingivalis*[37] (IMG gene id: 2599801257). A conserved domain for the propeptide (pfam08126) and the highly conserved Cys-His catalytic diad[38] were identified, and lend support for the presence of an unusual secreted protease from '*Ca.* Kryptonia' that could be active given the circumneutral pH found within the geothermal spring habitat.

CAzymes

Similar to that of *Bacteroidetes* and thermophilic sister phylum *Ignavibacteria*, the '*Ca.* Kryptonia' genomes harbor an expanded repertoire of glycosyl hydrolases, predicted enzymes containing carbohydrate-binding modules (CBMs), and other saccharolytic machinery for polysaccharide processing and degradation (Supplementary Data 14). In line with these predictions, we identified a TonB-dependent porin (SusC-like protein) and a glycan-binding SusD-like protein that formed the SusC/D outer membrane transport system for the putative uptake of oligosaccharides[39]. The SusC/D transport system was conserved across the majority of the '*Ca.* Kryptonia' genomes, but notably lacking from Great Boiling Springs '*Ca.* Thermokryptus mobilis' GFM JGI-1.

A plethora of glycosyltransferases of varying substrate specificity were identified (Supplementary Data 14), with the vast majority comprising seven CAzy (http://www.cazy.org/) families and the largest number constituting family GT41 with specificity for peptide β-N-acetylglucosaminyltransferase[40]. Twenty-three glycosyltransferase family GT41 homologs were identified in GFMs JGI-3 and JGI-4, and genome-wide estimates of combined glycoside hydrolases (GHs) and polysaccharide lyases (PLs) yielded an average 53 GHs/PLs per genome, comparable to members of the *Bacteroidetes* found in marine habitats and the human gut capable of organic matter degradation[41].

Bacillithiol
Notably, the gene complement *bshA* (glycosyltransferase), *bshB* (*N*-acetylhydrolase), and *bshC* (cysteine-adding enzyme) for the synthesis of bacillithiol, a major low-molecular-weight thiol in *Bacillus subtilis* and related bacteria[42], were identified across all the '*Ca.* Kryptonia' genotypes. Recently, bacillithiol has been found to contribute to oxidative stress resistance in *Staphylococcus aureus*[43]. In this context, the genetic potential for bacillithiol synthesis in '*Ca.* Kryptonia' might confer a functionally similar trait as in *S. aureus* and provide a protective role against reactive oxygen species. Additionally, we identified homologs for phytoene desaturase (*crtI*), beta-carotene 3-hydroxylase (*crtZ*), and a fusion type lycopene β-cyclase[44] for the biosynthesis of zeaxanthin glycosides. Curiously, the genetic potential for carotenoid biosynthesis was restricted to GFM JGI-3 from Gongxiaoshe pool, Yunnan Province, China and the complement SAGs including divergent genotype JGI-24.

Ether-linked membrane lipids
Unusually, we identified two geranylgeranylglyceryl phosphate synthases, which catalyze the first step in the synthesis of ether-linked membrane lipids in archaea, and a geranylgeranyl reductase suggestive of the incorporation of isoprendoid lipids into their membrane. The '*Ca.* Kryptonia' enzymes had close matches to clade IIb, and displayed homology to those previously identified within the *Bacteroidetes* with confirmed activity based on a radiolabelled substrate assay[45]. However, as with members of the *Bacteroidetes*, the '*Ca.* Kryptonia' genomes do not encode an *sn*-glycerol-1-phosphate dehydrogenase gene and therefore the presumptive function for the predicted geranylgeranylglyceryl phosphate synthases remains enigmatic.

Supplementary Note 3. Co-occurrence of '*Ca.* Kryptonia' with other lineages in shotgun metagenomic data
Since many standard primers used in SSU rRNA surveys of microbial community composition are biased against '*Ca.* Kryptonia,' we used SSU rRNA profiles obtained from shotgun metagenomic data for 22 geothermal sites (Supplementary Table 5) to assess co-occurrence of '*Ca.* Kryptonia' genotypes with other lineages and attempt to identify its metabolic partners. Mapping of raw reads to 16S and 18S rRNA sequences and their assembly retrieved 1,579 sequences longer than 300 nt. Since many of them contained introns, these sequences were trimmed to retain only nucleotides aligning to the 16S rRNA covariance model and clustered with similarly trimmed reference 16S sequences retrieved from the SILVA database. Clustering by UCLUST at 90%, 92% and 94% identity resulted in 136, 152 and 171 clusters, respectively, which had representatives from two or more hot spring samples. Computation of Spearman's rank-order correlation showed that the clusters most strongly correlated with '*Ca.* Kryptonia' did not change depending on the clustering cutoff; therefore 90% identity clusters were used in the subsequent analysis. 16S rRNA clusters at 90% identity most highly correlated with the abundance of '*Ca.* Kryptonia' genotypes listed in Supplementary Table 6 include *Armatimonadetes*, 3 different lineages of *Chloroflexi* and *Thermus* spp.

Supplementary Note 4. Reconstruction of the genome of *Armatimonadetes* lineage highly correlated with '*Ca.* Kryptonia' genotypes

Based on SSU rRNA analysis, the lineage of *Armatimonadetes* highly correlated with the presence of '*Ca.* Kryptonia' genotypes is very distant from publicly available *Armatimonadetes* genomes; therefore their genetic content is a poor predictor of possible metabolic interaction between the two populations. Phylogenetic placement of the *Armatimonadetes* was within Group 10, a group occurring exclusively in geothermal environments and ostensibly highly specialized[46]. In order to elucidate the relationship between these lineages, we identified metagenome contigs harboring SSU rRNA from *Armatimonadetes* cluster and used ESOM to assign additional contigs to this bin, recruit raw metagenomic reads and reassemble the genomes. The three reconstructed genomes were, on average, 2.82 Mb (± 0.12 s.d.) with an average 92.12% (± 1.22% s.d.) estimated genome completeness (Supplementary Table 3). Analysis of the reconstructed genome identified metabolic features complementary to those of '*Ca.* Kryptonia,' such as histidine (draft_100005467, draft_100005468, draft_100005469, draft_100012136, draft_100018113, draft_10001817, draft_100033510, draft_10003435), cysteine and methionine (draft_100002238, draft_1000045151, draft_100005413, draft_10000546, draft_10000936, draft_10000937, draft_100012363) and thiamine (draft_100008333, draft_10003351, draft_10003352) biosynthesis, and degradation of pentoses (draft_1000054113, draft_100006057, draft_10001037). Remarkably, in the reconstructed genomes we also identified a CsgG family protein (exemplified by draft_100006666). CsgG family proteins form transmembrane channels for secretion of "functional amyloids," a class of bacterial proteins capable of assembling highly stable fibers through a nucleation-precipitation mechanism[47]. "Functional amyloids" play major role in adhesion to surfaces and biofilm formation in diverse bacteria including *Escherichia coli*, *Caulobacter crescentus* and *Bacillus subtilis*[48]. CsgG-like transporter is found in a six-gene cluster conserved in reconstructed *Armatimonadetes* genomes. This cluster also includes a predicted subtilase-family peptidase (GxsBSedJan11_10003147), 3 hypothetical proteins of unknown function and putative 1800 amino acid secreted protein (GxsBSedJan11_10003145). The latter is characterized by the presence of four copies of so-called "carboxypeptidase regulatory-like domain" (PF13620). This domain is a member of the transthyretin clan and has been found to form amyloid in physiological conditions[49]. We hypothesize that the cluster in the *Armatimonadetes* genomes codes for synthesis, secretion and assembly of "functional amyloid", in which other members of the community may be embedded. On the other hand, '*Ca.* Kryptonia,' which harbor many proteases and peptidases in their genomes (see above), may be responsible for remodeling and digestion of this extracellular matrix.

Supplementary Note 5. '*Ca.* Kryptonia' potential to complement denitrification pathway in *Thermus* spp.

Thermus spp. have been experimentally characterized to reduce nitrate to nitrous oxide but lack the capacity to subsequently produce dinitrogen[13,50]. We found that '*Ca.* Kryptonia' encodes a nitrous oxide reductase (EC 1.7.2.4), which might complement the incomplete denitrification pathway in *Thermus* spp. (Supplementary Table 7). '*Ca.* Kryptonia' lacks other components of the denitrification pathway. The role of periplasmic

nitrate reductase, Nap, in '*Ca.* Kryptonia' is less clear. In most organisms, this enzyme functions in dissimilatory nitrate reduction to ammonia, a pathway thought to be more favorable under conditions of low nitrate concentration, but high electron donor availability[51]. So while Thermus spp. may be responsible for denitrification at high nitrate concentrations, '*Ca.* Kryptonia' may take over as a nitrate reducer at lower nitrate levels. However, in other organisms, periplasmic nitrate reductase alone is capable of supporting anaerobic growth[52]. Further, unlike bacterial respiratory nitrate reductase and assimilatory nitrate reductase, the active center of Nap is located in the periplasm, so no transmembrane nitrate/nitrite exchange is necessary[53]. Nitrous oxide does not require any transporters to reach the cytosol; but in any case nitrous oxide reductases are periplasmic or extracellular enzymes.

Supplementary References

1. Stamatakis, A. RAxML-VI-HPC: maximum likelihood-based phylogenetic analyses with thousands of taxa and mixed models. *Bioinformatics* **22**, 2688-2690 (2006).
2. Castelle, C. J. *et al.* Extraordinary phylogenetic diversity and metabolic versatility in aquifer sediment. *Nat. Commun.* **4** (2013).
3. Alkhnbashi, O. S. *et al.* CRISPRstrand: predicting repeat orientations to determine the crRNA-encoding strand at CRISPR loci. *Bioinformatics* **30**, i489-496 (2014).
4. Lange, S. J., Alkhnbashi, O. S., Rose, D., Will, S. & Backofen, R. CRISPRmap: an automated classification of repeat conservation in prokaryotic adaptive immune systems. *Nucleic Acids Res.* **41**, 8034-8044 (2013).
5. Burnouf, D. Y. *et al.* Structural and biochemical analysis of sliding clamp/ligand interactions suggest a competition between replicative and translesion DNA polymerases. *J. Mol. Biol.* **335**, 1187-1197 (2004).
6. Timinskas, K., Balvočiūtė, M., Timinskas, A. & Venclovas, Č. Comprehensive analysis of DNA polymerase III α subunits and their homologs in bacterial genomes. *Nucleic Acids Res.* **42**, 1393-1413 (2014).
7. Bressanin, D. *et al.* Proteolysis of the proofreading subunit controls the assembly of *Escherichia coli* DNA polymerase III catalytic core. *Biochim. Biophys. Acta* **1794**, 1606-1615 (2009).
8. Jeruzalmi, D., O'Donnell, M. & Kuriyan, J. Crystal structure of the processivity clamp loader gamma (γ) complex of *E. coli* DNA polymerase III. *Cell* **106**, 429-441 (2001).
9. Peacock, J. P. *et al.* Pyrosequencing reveals high-temperature cellulolytic microbial consortia in Great Boiling Spring after *in situ* lignocellulose enrichment. *PLoS One* **8**, e59927 (2013).
10. Hou, W. *et al.* A comprehensive census of microbial diversity in hot springs of Tengchong, Yunnan Province China using 16S rRNA gene pyrosequencing. *PLoS One* **8** (2013).
11. Sharp, C. E. *et al.* Humboldt's spa: microbial diversity is controlled by temperature in geothermal environments. *ISME J.* **8**, 1166-1174 (2014).

12. Yarza, P. *et al.* Uniting the classification of cultured and uncultured bacteria and archaea using 16S rRNA gene sequences. *Nature Rev. Microbiol.* **12**, 635-645 (2014).
13. Murugapiran, S. K. *et al. Thermus oshimai* JL-2 and *T. thermophilus* JL-18 genome analysis illuminates pathways for carbon, nitrogen, and sulfur cycling. *Stand. Genomic Sci.* **7**, 449-468 (2013).
14. Haft, D. H., Selengut, J., Mongodin, E. F. & Nelson, K. E. A guild of 45 CRISPR-associated (Cas) protein families and multiple CRISPR/Cas subtypes exist in prokaryotic genomes. *PLoS Comp. Biol.* **1**, e60 (2005).
15. Zhang, Q., Doak, T. G. & Ye, Y. Expanding the catalog of cas genes with metagenomes. *Nucleic Acids Res.* **42**, 2448-2459 (2014).
16. Makarova, K. S. *et al.* Evolution and classification of the CRISPR-Cas systems. *Nature Rev. Microbiol.* **9**, 467-477 (2011).
17. van der Oost, J., Westra, E. R., Jackson, R. N. & Wiedenheft, B. Unravelling the structural and mechanistic basis of CRISPR-Cas systems. *Nature Rev. Microbiol.* **12**, 479-492 (2014).
18. Koonin, E. V. & Makarova, K. S. CRISPR-Cas: evolution of an RNA-based adaptive immunity system in prokaryotes. *RNA Biol.* **10**, 679-686 (2013).
19. Makarova, K. S., Wolf, Y. I. & Koonin, E. V. The basic building blocks and evolution of CRISPR-CAS systems. *Biochem. Soc. Trans.* **41**, 1392-1400 (2013).
20. Skennerton, C. T., Imelfort, M. & Tyson, G. W. Crass: identification and reconstruction of CRISPR from unassembled metagenomic data. *Nucleic Acids Res.* **41**, e105 (2013).
21. Lucchini, S., Desiere, F. & Brüssow, H. Comparative genomics of *Streptococcus thermophilus* phage species supports a modular evolution theory. *J. Virol.* **73**, 8647-8656 (1999).
22. Inskeep, W. P. *et al.* The YNP metagenome project: Environmental parameters responsible for microbial distribution in the Yellowstone geothermal ecosystem. *Front. Microbiol.* **4**, 67 (2013).
23. Heine, M. & Chandra, S. C. The linkage between reverse gyrase and hyperthermophiles: A review of their invariable association. *J. Microbiol.* **47**, 229-234 (2009).
24. Brochier-Armanet, C. & Forterre, P. Widespread distribution of archaeal reverse gyrase in thermophilic bacteria suggests a complex history of vertical inheritance and lateral gene transfers. *Archaea* **2**, 83-93 (2007).
25. Kimura, H., Sugihara, M., Kato, K. & Hanada, S. Selective phylogenetic analysis targeted at 16S rRNA genes of thermophiles and hyperthermophiles in deep-subsurface geothermal environments. *Appl. Environ. Microbiol.* **72**, 21-27 (2006).
26. Kasprzak, J. M., Czerwoniec, A. & Bujnicki, J. M. Molecular evolution of dihydrouridine synthases. *BMC Bioinformatics* **13**, 153-153 (2012).
27. Noon, K. R. *et al.* Influence of temperature on tRNA modification in Archaea: *Methanococcoides burtonii* (Optimum Growth Temperature [T_{opt}], 23°C) and *Stetteria hydrogenophila* (T_{opt}, 95°C). *J. Bacteriol.* **185**, 5483-5490 (2003).
28. Hori, H. Methylated nucleosides in tRNA and tRNA methyltransferases. *Front. Genet.* **5**, 144 (2014).

29. Persson, B. C., Jäger, G. & Gustafsson, C. The spoU gene of *Escherichia coli*, the fourth gene of the spoT operon, is essential for tRNA (Gm18) 2'-O-methyltransferase activity. *Nucleic Acids Res.* **25**, 4093-4097 (1997).
30. Purta, E. *et al.* The yfhQ gene of *Escherichia coli* encodes a tRNA:Cm32/Um32 methyltransferase. *BMC Mol. Biol.* **7**, 23-23 (2006).
31. Benítez-Páez, A., Villarroya, M., Douthwaite, S., Gabaldón, T. & Armengod, M. E. YibK is the 2'-O-methyltransferase TrmL that modifies the wobble nucleotide in *Escherichia coli* tRNA(Leu) isoacceptors. *RNA* **16**, 2131-2143 (2010).
32. Empadinhas, N. & da Costa, M. S. Diversity and biosynthesis of compatible solutes in hyperthermophiles. *Int. Microbiol.* **9**, 199-206 (2006).
33. Borges, N., Ramos, A., Raven, N., Sharp, R. & Santos, H. Comparative study of the thermostabilizing properties of mannosylglycerate and other compatible solutes on model enzymes. *Extremophiles* **6**, 209-216 (2002).
34. Bryant, D. A. & Frigaard, N.-U. Prokaryotic photosynthesis and phototrophy illuminated. *Trends Microbiol.* **14**, 488-496 (2006).
35. McCutcheon, J. P. & Moran, N. A. Parallel genomic evolution and metabolic interdependence in an ancient symbiosis. *Proc. Natl. Acad. Sci. U.S.A.* **104**, 19392-19397 (2007).
36. Robinson, A., Causer, R. J. & Dixon, N. E. Architecture and conservation of the bacterial DNA replication machinery, an underexploited drug target. *Curr. Drug Targets* **13**, 352-372 (2012).
37. Sato, K. *et al.* A protein secretion system linked to bacteroidete gliding motility and pathogenesis. *Proc. Natl. Acad. Sci. U.S.A.* **107**, 276-281 (2010).
38. Eichinger, A. *et al.* Crystal structure of gingipain R: an Arg-specific bacterial cysteine proteinase with a caspase-like fold. *EMBO J.* **18**, 5453-5462 (1999).
39. Bolam, D. N. & Koropatkin, N. M. Glycan recognition by the *Bacteroidetes* Sus-like systems. *Curr. Opin. Struct. Bio.* **22**, 563-569 (2012).
40. Lombard, V., Golaconda Ramulu, H., Drula, E., Coutinho, P. M. & Henrissat, B. The carbohydrate-active enzymes database (CAZy) in 2013. *Nucleic Acids Res.* **42**, D490-D495 (2014).
41. Thomas, F., Hehemann, J.-H., Rebuffet, E., Czjzek, M. & Michel, G. Environmental and gut *Bacteroidetes*: the food connection. *Front. Microbiol.* **2**, 93 (2011).
42. Gaballa, A. *et al.* Biosynthesis and functions of bacillithiol, a major low-molecular-weight thiol in *Bacilli*. *Proc. Natl. Acad. Sci. U.S.A.* **107**, 6482-6486 (2010).
43. Posada, A. C. *et al.* Importance of bacillithiol in the oxidative stress response of *Staphylococcus aureus*. *Infect. Immun.* **82**, 316-332 (2014).
44. H Hemmi, H., Ikejiri, S., Nakayama, T. & Nishino, T. Fusion-type lycopene β-cyclase from a thermoacidophilic archaeon *Sulfolobus solfataricus*. *Biochem. Biophys. Res. Commun.* **305**, 586-591 (2003).
45. Peterhoff, D. *et al.* A comprehensive analysis of the geranylgeranylglyceryl phosphate synthase enzyme family identifies novel members and reveals mechanisms of substrate specificity and quaternary structure organization. *Mol. Microbiol.* **92**, 885-899 (2014).
46. Lee, K. Y., Dunfield, P. & Stott, M. in *The Prokaryotes* (eds Rosenberg *et al.*) Ch. 32 The phylum *Armatimonadetes*, 447-458 (Springer Berlin Heidelberg, 2014).

47. Cao, B. *et al.* Structure of the nonameric bacterial amyloid secretion channel. *Proc. Natl. Acad. Sci. U.S.A.* **111**, E5439-E5444 (2014).
48. Evans, M. L. & Chapman, M. R. Curli biogenesis: order out of disorder. *Biochim. Biophys. Acta* **1843**, 1551-1558 (2014).
49. Garcia-Pardo, J. *et al.* Amyloid formation by human carboxypeptidase D transthyretin-like domain under physiological conditions. *J. Biol. Chem.* **289**, 33783-33796 (2014).
50. Hedlund, B. P. *et al.* Potential role of *Thermus thermophilus* and *T. oshimai* in high rates of nitrous oxide (N_2O) production in ~80°C hot springs in the US Great Basin. *Geobiology* **9**, 471-480 (2011).
51. Christensen, P. B., Rysgaard, S. r., Sloth, N. P., Dalsgaard, T. & Schwærter, S. Sediment mineralization, nutrient fluxes, denitrification and dissimilatory nitrate reduction to ammonium in an estuarine fjord with sea cage trout farms. *Aquat. Microb. Ecol.* **21**, 73-84 (2000).
52. Li, Y., Katzmann, E., Borg, S. & Schüler, D. The periplasmic nitrate reductase Nap is required for anaerobic growth and involved in redox control of magnetite biomineralization in *Magnetospirillum gryphiswaldense*. *J. Bacteriol.* **194**, 4847-4856 (2012).
53. Kraft, B., Strous, M. & Tegetmeyer, H. E. Microbial nitrate respiration – Genes, enzymes and environmental distribution. *J. Biotechnol.* **155**, 104-117 (2011).
